# Elevational distribution and seasonal dynamics of alpine soil prokaryotic communities

**DOI:** 10.3389/fmicb.2023.1280011

**Published:** 2023-09-22

**Authors:** Junpeng Rui, Yuwei Zhao, Nan Cong, Fuxin Wang, Chao Li, Xiang Liu, Jingjing Hu, Ning Ling, Xin Jing

**Affiliations:** ^1^State Key Laboratory of Herbage Improvement and Grassland Agro-ecosystems, College of Ecology, Lanzhou University, Lanzhou, China; ^2^Center for Grassland Microbiome, College of Pastoral Agriculture Science and Technology, Lanzhou University, Lanzhou, China; ^3^Lhasa Plateau Ecosystem Research Station, Institute of Geographic Sciences and Natural Resources Research, Chinese Academy of Sciences, Beijing, China; ^4^State Key Laboratory of Herbage Improvement and Grassland Agro-ecosystems, College of Pastoral Agriculture Science and Technology, Lanzhou University, Lanzhou, China

**Keywords:** soil microbial community, altitudinal gradient, temporal dynamic, alpine grassland, Actinobacteria, community assembly

## Abstract

The alpine grassland ecosystem is a biodiversity hotspot of plants on the Qinghai-Tibetan Plateau, where rapid climate change is altering the patterns of plant biodiversity along elevational and seasonal gradients of environments. However, how belowground microbial biodiversity changes along elevational gradient during the growing season is not well understood yet. Here, we investigated the elevational distribution of soil prokaryotic communities by using 16S rRNA amplicon sequencing along an elevational gradient between 3,200 and 4,200 m, and a seasonal gradient between June and September in the Qinghai-Tibetan alpine grasslands. First, we found soil prokaryotic diversity and community composition significantly shifted along the elevational gradient, mainly driven by soil temperature and moisture. Species richness did not show consistent elevational trends, while those of evenness declined with elevation. Copiotrophs and symbiotic diazotrophs declined with elevation, while oligotrophs and AOB increased, affected by temperature. Anaerobic or facultatively anaerobic bacteria and AOA were hump-shaped, mainly influenced by moisture. Second, seasonal patterns of community composition were mainly driven by aboveground biomass, precipitation, and soil temperature. The seasonal dynamics of community composition indicated that soil prokaryotic community, particularly Actinobacteria, was sensitive to short-term climate change, such as the monthly precipitation variation. At last, dispersal limitation consistently dominated the assembly process of soil prokaryotic communities along both elevational and seasonal gradients, especially for those of rare species, while the deterministic process of abundant species was relatively higher at drier sites and in drier July. The balance between deterministic and stochastic processes in abundant subcommunities might be strongly influenced by water conditions (precipitation/moisture). Our findings suggest that both elevation and season can alter the patterns of soil prokaryotic biodiversity in alpine grassland ecosystem of Qinghai-Tibetan Plateau, which is a biodiversity hotspot and is experiencing rapid climate change. This work provides new insights into the response of soil prokaryotic communities to changes in elevation and season, and helps us understand the temporal and spatial variations in such climate change-sensitive regions.

## Introduction

As hotspots of biodiversity and climate change, mountains create a broad range of gradients in both abiotic and biotic settings (Martin and Bellingham, 2016), and provide ecologists with a natural setting for testing their hypotheses (Lomolino, 2001). Spatial distributions of flora and animals along elevational gradients have been extensively studied (Rahbek, 1995). The most prevalent elevational pattern is a hump-shaped species-richness pattern for plants and animals, followed by a decreasing pattern of species richness (Rahbek, 2005). However, the elevational patterns for soil microorganisms are not well understood that may not be the same as those of animals and plants (Fierer et al., 2011). Generally, elevational patterns of soil microbes are diverse and inconclusive, including five types (type I-V): increase, decrease, hump-shaped, U-shaped, and no discernable tread (Hendershot et al., 2017; Looby and Martin, 2020).

Many environmental factors change along elevation since it is a complex and indirect gradient (Fierer et al., 2011). For example, temperature declines with increased elevation (Körner, 2007), coupled with decreased aboveground biomass (Hendershot et al., 2017), and while precipitation is often hump-shaped because of the cloud zone at mid-elevation (Rahbek, 1995). Consequently, various environmental settings, including temperature, precipitation, vegetation, and soil characteristics, may have an impact on the diversity and community composition of soil microbes (Sundqvist et al., 2013; Singh et al., 2014; Nottingham et al., 2018). However, key factors driving the elevational distribution are still under debate. The key factor may be different owing to different vegetation types or sampling scales on the same mountain. For instance, soil pH drives bacterial community distribution along six elevations between 530 m and 2,200 m a.s.l. on Changbai Mountain (Shen et al., 2013), while the primary determinants of bacterial elevational distribution are soil carbon and nitrogen concentrations at a rather small scale from 2000 m to 2,500 m a.s.l. on the same mountain (Shen et al., 2015).

Environmental factors, including temperature, precipitation, vegetation, and soil characteristics, may change with seasons, resulting in seasonal dynamics of the soil microbes (Lazzaro et al., 2015). For example, since different soil microbial taxa may differ in their response to changes in soil moisture and temperature, the seasonal dynamics of soil temperature and moisture strongly influence the abundance of soil bacteria in a desert grassland (Bell et al., 2009). A study of the alpine ecosystem demonstrates that Acidobacteria was more abundant in spring, Actinobacteria and Bacteroides in winter, while Verrucomicrobia and Betaproteobacteria in summer (Lipson and Schmidt, 2004). Alpine ecosystems go through a remarkable variety of seasonal variations (Ernakovich et al., 2014). However, few studies have focused on seasonal dynamics of soil microbial communities along elevational gradients. According to a study along the tree line, elevational effects on soil microbial diversity outweigh seasonal influences (Shen et al., 2021). At every elevation, the bacterial and fungal community structures exhibit an annual cycle in the catchment of the Tiefen Glacier (Lazzaro et al., 2015).

The community assembly mechanism is a critical issue in microbial ecology (Stegen et al., 2012). Stochastic processes include homogenous dispersal, dispersal limitation, and drift, while deterministic processes consist of homogenous selection and heterogeneous selection (Stegen et al., 2015; Shi et al., 2022). Some studies reported that deterministic processes play critical roles in bacterial community assembly along elevation gradients in some sites of the Qinghai-Tibetan Plateau, where the range of soil pH was broad (Li et al., 2018; Shen et al., 2019). However, a transect study across the Qinghai-Tibetan Plateau showed that stochastic processes, notably dispersal limitation and drift, played a primary role in bacterial community assembly in alpine meadow soils (Kang et al., 2022).

Alpine grasslands, the dominant vegetation on the Qinghai-Tibetan Plateau, are fragile and sensitive to climate change. Soil microorganisms are mainly concentrated in surface soils of this area, and play important roles in C and N cycles (Zhao and Zhou, 1999). The mechanism of soil microbial distribution along an elevation gradient in alpine grasslands keeps unclear. Precipitation is a primary driving force on the bacterial community between 4,400 and 5,200 m a.s.l. over the Qinghai-Tibetan alpine grasslands at Damxung station (Yuan et al., 2014), while temperature may be more significant at Haibei station, Qinghai (Rui et al., 2015). Besides, the seasonal dynamics of soil microbial community along elevation may occur since climate, soil properties, and microbial biomass are seasonal various (Chen et al., 2021).

In this study, we aim to elucidate the elevational distribution and seasonal dynamics of soil prokaryotic communities along an elevational gradient (3200–4,200 m) in the Qinghai-Tibetan alpine grassland during the growing season. The main goal of this study is to answer the following questions: (i) What is the critical factor driving the elevational distribution of soil prokaryotic community? (ii) Whether soil prokaryotic community significantly changes during the growing season? and (iii) Whether the ecological processes of prokaryotic community assembly change along the elevational or seasonal gradients?

## Materials and methods

### Study site description and experimental design

The study site is located at the Haibei National Alpine Grassland Ecosystem Research Station (101°12′E, 37°37’N, ~ 3,200 m above sea level) on the northeastern Qinghai-Tibetan Plateau in Qinghai, China. The annual mean air temperature is −1.1°C, and the annual mean precipitation is 485 mm (Ma et al., 2017). The soil is classified as Mat-Gryic Cambisol, and is dominated by perennial plants (Ma et al., 2017). The growing season lasts from May to September, when more than 80% of the annual rainfall falls (Yang et al., 2014).

Study sites were located at six elevations along an elevational gradient (3,200, 3,400, 3,600, 3,800, 4,000, and 4,200 m). Six 1 m × 1 m quadrats were selected as replicates at each elevation. The geographic coordinates and vegetation information for each elevation were reported previously (Rui et al., 2022). We collected soil samples at the beginning of June, July, August, and September in 2021, respectively. Five soil cores (5 cm in diameter) with 0–10 cm depth were taken randomly within each quadrat, mixed as one sample, and sieved with a 2 mm mesh. The aboveground plant biomass and richness were surveyed at the same time (Supplementary Figure S1). Soil temperature was monitored using EM-50 devices (Decagon devices, United States) at 5 cm, and the mean monthly temperature was calculated. Methods of measuring soil moisture, pH, total nitrogen (TN), total organic carbon (TOC), nitrate, nitrite, and ammonium contents were reported previously (Rui et al., 2022). Monthly precipitations at 3200 m between June and September in 2021 were 90, 64, 125, and 104 mm, respectively. The data of soil and plant properties are in the Supplementary materials.

### DNA extraction, qPCR, and sequencing

PowerSoil DNA Isolation kit (MO BIO Laboratories, United States) was used to extract genomic DNA in soils following the manufacturer’s instructions (Rui et al., 2022). The universal primers 515F and 806R were used for the amplification of the 16S rRNA gene for qPCR and sequencing (Borton et al., 2017). The amplifications for sequencing were conducted following the “16S Illumina Amplicon Protocol” on the website of the Earth Microbiome Project.^1^ Real-time PCR was performed on the Applied Biosystems 7,300 Sequence Detection System (ABI, United States). The qPCR reaction system was composed of 10 μL of 2X Taq Plus Master Mix (Vazyme Biotech Co., Ltd., China), 0.8 μL of forward primer (5 μmol L^−1^), 0.8 μL of reverse primer, 1 μL of DNA, and 7.4 μL of ddH_2_O. The PCR program included an initial denaturation at 95°C for 5 min, followed by 35 cycles of 95°C for 30 s, 58°C for 30 s, and 72°C for 1 min. Sequencing libraries were generated with TruSeq® DNA Kit (Illumina, United States), and sequenced with the Reagent Kit v2 (2 × 250 bp) on the Illumina NovaSeq 6,000 platform (Novogene, Beijing, China) (Rui et al., 2022).

### Sequencing data analysis

FLASh v1.2.11 was used to merge the paired-end reads (Magoč and Salzberg, 2011). Primers were removed from the merged reads using the Perl script trim_primer_in fq.pl (All Perl scripts mentioned in this article are available on GitHub at https://github.com/PeterRui/perl4amplicon). Low-quality reads were filtered using Trimomatic version 0.39 (Bolger et al., 2014). Chimeras were checked using Usearch 11.0.667.^2^ Amplicon sequence variants (ASVs) were obtained using the Unoise3 algorithm (Edgar, 2016a). The representative reads of ASVs were aligned with PyNAST, and the phylogenetic tree was constructed with Qiime 1.9.1 (Caporaso et al., 2010). The taxonomy of ASV-representative sequences was predicted with the RDP training set v16 database using the SINTAX algorithm (Edgar, 2016b). Moreover, chloroplasts and mitochondria from eukaryotic cells were checked using Greengenes v13.5 and SILVA v123 database using SINTAX. Sequences of each sample in the ASV-table were randomly normalized to the same sequence depth using the Perl script subsample_in_table.pl. After subsample, a total of 3,947,600 high-quality 16S rRNA sequences were obtained from 142 samples, affiliated to 41,750 ASVs. Alpha diversity indices, such as observed species, Chao1 richness, Shannon diversity, reciprocal Simpson diversity (1/D), and Pielou evenness index were calculated with the script alpha_diversity.pl. The Supplementary material contains information on using Perl scripts.

### Statistical analyses

We performed statistical analyses based on the ASV-table and/or environmental factors using R (Version 4.1.0). Details of the statistical analysis can be found in a previous publication (Rui et al., 2022). For example, the function *varclus* in package *Hmisc* and *vif.cca* in package *vegan* were used to estimate collinearities among environmental factors. Using the function *HSD.test* in package *agricolae*, ANOVA with Tukey’s test was calculated to check the differences of prokaryotic groups or environmental factors among elevations/months. Bray-Curtis distance-based PCoA was conducted using *cmdscale* in *vegan*. Environmental factors were fitted onto the PCoA plots using *envfit* in *vegan*. Adonis (i.e., PerMANOVA) was estimated using *adonis* to evaluate community differences between sample groups. To calculate the multivariate homogeneity of community dispersions, PERMDISP was estimated using *betadisper*. Partial Mantel test and multiple regression on distance matrices (MRM) were conducted with the package *ecodist* to evaluate the effects of environmental factors on the prokaryotic community. The package *rdacca.hp* was used to estimate the independent contribution of each factor to prokaryotic community variation (Lai et al., 2022).

### Community assembly processes of subcommunities

Abundant and rare subcommunities may have different ecological roles and community assembly mechanisms (Logares et al., 2014; Jiao et al., 2017). The definition of abundant, intermediate, and rare species were modified based on Jiao’s method (Jiao and Lu, 2020). Specifically, across all samples, ASVs with average relative abundances <0.001% and > 0.01% were defined as rare and abundant species, respectively. Those between 0.001% and 0.01% were defined as intermediate species. The community assembly processes of abundant, intermediate, and rare species were evaluated with iCAMP, using the confidence index for the null model significance test. ASVs were divided into different “bins” based on the phylogenetic tree, and their ecological processes were identified using null model analysis (Ning et al., 2020).

## Results

### Elevational shift of prokaryotic diversity and community composition

**Figure 1:**
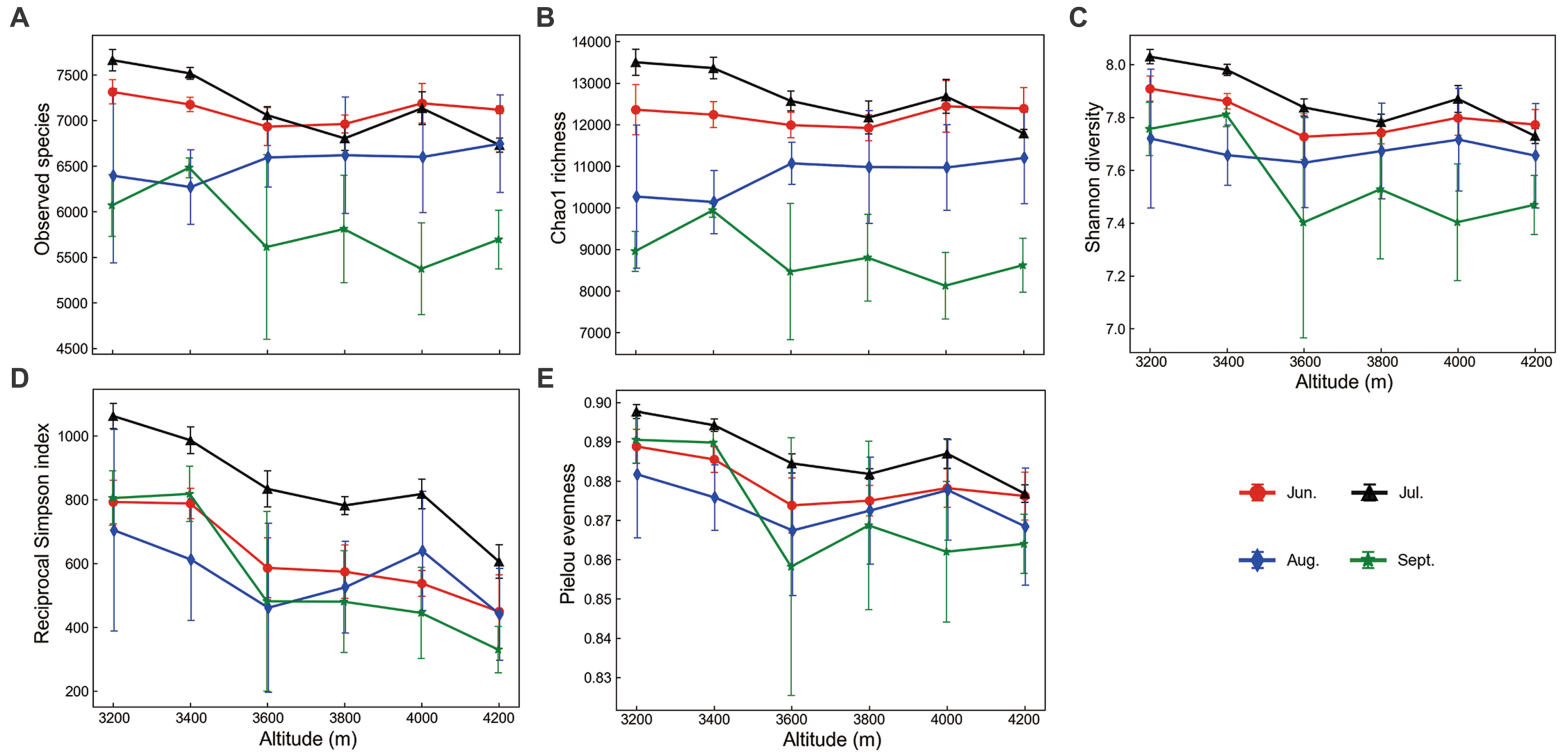
Elevational changes of prokaryotic alpha diversity indices during the growing season. Error bars represent standard deviations (*n* = 6).

Alpha diversity indices changed significantly along the elevational gradient (two-way ANOVA *p* < 0.001). In general, elevational changes of evenness were more obvious than that of species richness (Observed species and Chao1 index). Elevational patterns of species richness were almost no trend in most cases (Figures 1A,B), while those indices including Shannon diversity, reciprocal Simpson diversity, and Pielou evenness declined with elevation, although not always monotonic (Figures 1C–E). Soil temperature was the most important factor in elevational shift, positively correlated to alpha diversity, especially evenness (*p* < 0.01).

**Figure 2:**
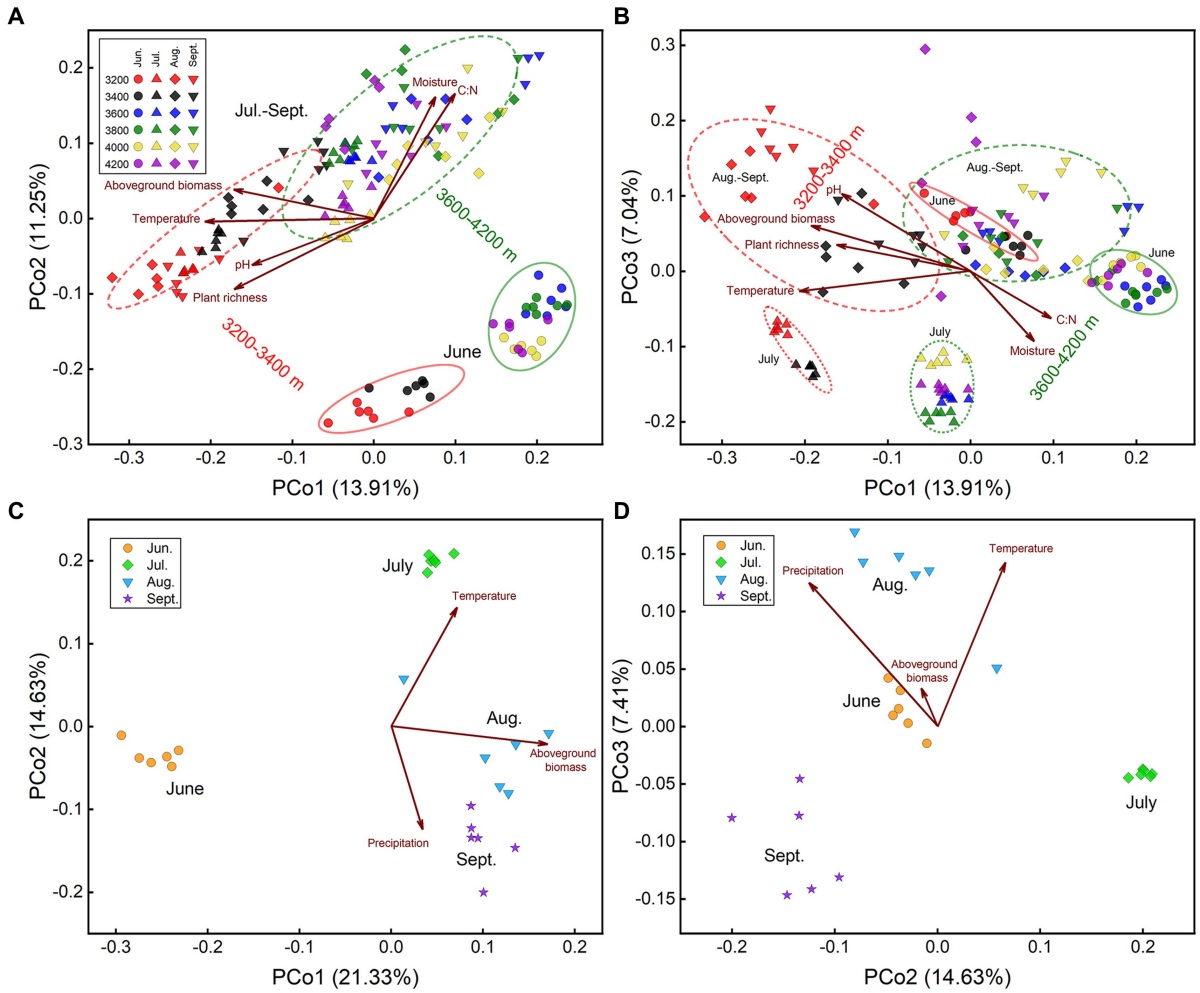
PCoA plots of prokaryotic community structure based on Bray-Curtis distance. Plots include all samples with **(A)** axis 1 vs. axis 2, and **(B)** with axis 1 vs. axis 3; samples at 3200 m **(C)** with axis 1 vs. 2, and **(D)** with axis 2 vs. 3, respectively. Important environmental factors were fitted onto the patterns. Values on axes indicated the percentages of total variation explained by each axis.

The community structures at low elevations (3200–3,400 m) were substantially different from those at high elevations (3600–4,200 m), which could be distinguished along axis 1 of PCoA (Figure 2A). In the same month, most elevations we sampled had substantially distinct prokaryotic community structures (Supplementary Figure S2). Soil temperature was the critical factor driving the prokaryotic elevational distribution in each month (Tables 1–3). Temperature was strongly correlated to prokaryotic community structure, best fitted in PCoA patterns in each month (*R*^2^ ≥ 0.75, Supplementary Figure S3). Moisture was another important factor to elevational distribution, particularly in July (Tables 1–3). The effects of temperature and moisture decreased in August, in which the community dispersions were higher (Supplementary Figure S4). In addition, soil pH was important to elevational distribution in June, while plant richness in July (Tables 1, 2). It seems that aboveground biomass was less important because of its collinearity with temperature. The impact of precipitation along the elevation gradient was unclear due to lack of data.

**Table 1:** Spearman’s correlation coefficients of prokaryotic community structure with environmental factors based on partial Mantel test.

	Ammonium	Nitrate	Nitrite	TN	C:N ratio	pH	Moisture	Precipitation	Temperature	Plant richness	Plant biomass
All	0.050	0.044	0.012	−0.002	−0.076^*^	0.094^*^	0.100^***^	n.a.	**0.144** ^***^	0.078^**^	**0.246** ^***^
3,200 m	−0.089	−0.025	0.089	0.005	0.012	−0.002	0.018	0.160^**^	0.238^**^	0.037	**0.664** ^***^
3,400 m	−0.002	0.003	0.062	−0.167	−0.160	0.166	−0.028	n.a.	**0.296** ^***^	−0.035	**0.520** ^***^
3,600 m	−0.169	−0.110	−0.144	−0.109	0.156	−0.003	−0.035	n.a.	**0.432** ^***^	−0.101	0.168
3,800 m	0.156	0.258^**^	a	0.085	0.003	0.013	0.111	n.a.	0.014	−0.054	0.006
4,000 m	0.011	−0.024	−0.079	0.049	0.158	0.071	−0.035	n.a.	**0.432** ^***^	−0.044	−0.107
4,200 m	0.324^*^	0.043	−0.185	−0.074	0.045	0.112	−0.010	n.a.	0.114	−0.158	0.280^*^
June	−0.083	0.067	−0.017	0.174^**^	0.104	**0.346** ^***^	**0.211** ^***^	n.a.	**0.579** ^***^	0.140^*^	−0.125^*^
July	0.106^*^	0.000	−0.012	0.121^*^	−0.110^*^	0.054	**0.568** ^***^	n.a.	**0.776** ^***^	**0.258** ^***^	0.018
August	−0.073	0.060	−0.170^*^	−0.067	0.146	0.142	−0.057	n.a.	**0.313** ^***^	0.075	0.087
September	0.027	−0.125	0.069	b	0.009	0.161^**^	**0.341** ^***^	n.a.	**0.295** ^***^	−0.023	0.038

When one environmental factor was analyzed by partial analyses, the remaining environmental variables were controlled for. n.a.: not applicable due to lack of precipitation data at different elevations. Significance: ^*^p < 0.05. ^**^p < 0.01. ^***^p < 0.001 (values in bold). a. Nitrite was strongly collinear with temperature (Pearson’s r^2^ > 0.7). b. TN was strongly collinear with moisture (Pearson’s r^2^ > 0.7).

**Table 2:** MRM regression coefficients of prokaryotic community structure with environmental factors.

	Ammonium	Nitrate	Nitrite	TN	C:N ratio	pH	Moisture	Precipitation	Temperature	Plant richness	Plant biomass
All	−0.048	−0.039	−0.011	0.002	−0.074^*^	−0.101^*^	−0.104^**^	n.a.	**−0.147** ^***^	−0.079^**^	**−0.250** ^***^
3,200 m	0.063	0.019	−0.064	−0.003	−0.008	0.001	−0.013	−0.214^**^	−0.235^**^	−0.025	**−0.676** ^***^
3,400 m	0.002	−0.002	−0.049	0.135	0.120	−0.138	0.021	n.a.	−0.272^**^	0.026	**−0.502** ^***^
3,600 m	0.154	0.100	0.122	0.094	−0.134	0.003	0.029	n.a.	**−0.517** ^***^	0.086	−0.166
3,800 m	−0.154	−0.317^*^	a	−0.083	−0.002	−0.013	−0.108	n.a.	−0.022	0.052	−0.008
4,000 m	−0.010	0.025	0.077	−0.046	−0.153	−0.064	0.033	n.a.	**−0.508** ^***^	0.042	0.103
4,200 m	−0.307^*^	−0.041	0.166	0.067	−0.041	−0.099	0.009	n.a.	−0.111	0.148	−0.273^*^
June	0.067	−0.038	0.010	−0.116^**^	−0.061	**−0.215** ^***^	−0.129^**^	n.a.	**−0.706** ^***^	−0.097^*^	0.072^*^
July	−0.052^*^	0.000	0.005	−0.055^*^	0.053^*^	−0.030	**−0.330** ^***^	n.a.	**−0.715** ^***^	**−0.131** ^***^	−0.008
August	0.066	−0.052	0.155^*^	0.072	−0.130	−0.157	0.062	n.a.	**−0.365** ^***^	−0.073	−0.087
September	−0.029	0.102	−0.055	b	−0.009	−0.170^*^	**−0.294** ^***^	n.a.	**−0.400** ^***^	0.022	−0.042

n.a.: not applicable due to lack of precipitation data at different elevations. Significance: ^*^p < 0.05. ^**^p < 0.01. ^***^p < 0.001 (values in bold).a. Nitrite was strongly collinear with temperature (Pearson’s r^2^ > 0.7).b. TN was strongly collinear with moisture (Pearson’s r^2^ > 0.7).

**Table 3:** The independent contribution of each factor to prokaryotic community variation based on hierarchical partitioning in canonical analysis.

	Ammonium	Nitrate	Nitrite	TN	C: N ratio	pH	Moisture	Precipitation	Temperature	Plant richness	Plant biomass
All	0.023^**^	0.017^**^	0.012	0.017^**^	0.016^**^	0.022^*^	**0.028** ^***^	n.a.	**0.048** ^***^	0.024^**^	**0.043** ^***^
3,200 m	0.012	0.026	0.030	0.008	0.001	0.016	0.035	0.057^*^	0.060^*^	0.001	0.078^*^
3,400 m	0.014	0.021	0.007	0.022	−0.006	0.016	0.012	n.a.	0.055	−0.008	0.093^**^
3,600 m	−0.002	0.048^*^	0.030^*^	−0.005	0.027	0.000	0.018	n.a	0.076^*^	−0.008	0.048
3,800 m	0.016	0.033	a	0.024	0.050^*^	−0.004	0.052	n.a	0.051	0.001	0.021
4,000 m	−0.002	0.018	0.014	0.012	0.013	−0.001	0.004	n.a.	0.061	−0.004	0.024
4,200 m	−0.010	0.015	−0.012	0.015	0.031^*^	0.005	0.003	n.a.	0.073^*^	−0.011	0.097^**^
June	0.035	0.006	0.006^*^	0.026^**^	0.009	0.031	**0.042** ^***^	n.a.	**0.060** ^***^	0.033	0.013
July	0.029	0.018	0.005	0.021^*^	0.022^**^	0.035^*^	**0.054** ^***^	n.a.	**0.074** ^***^	0.037	0.015
August	0.022	0.006	0.006	−0.001	0.004	0.023	0.010	n.a.	0.043	0.039	0.031
September	0.025	−0.005	0.016	b	0.033^*^	0.040	0.060^*^	n.a.	0.051^*^	0.018	0.034

n.a.: not applicable due to lack of precipitation data at different elevations. Significance: ^*^p < 0.05. ^**^p < 0.01. ^***^p < 0.001 (values in bold).a. Nitrite was strongly collinear with temperature (Pearson’s r^2^ > 0.7).b. TN was strongly collinear with moisture (Pearson’s r^2^ > 0.7).

**Figure 3:**
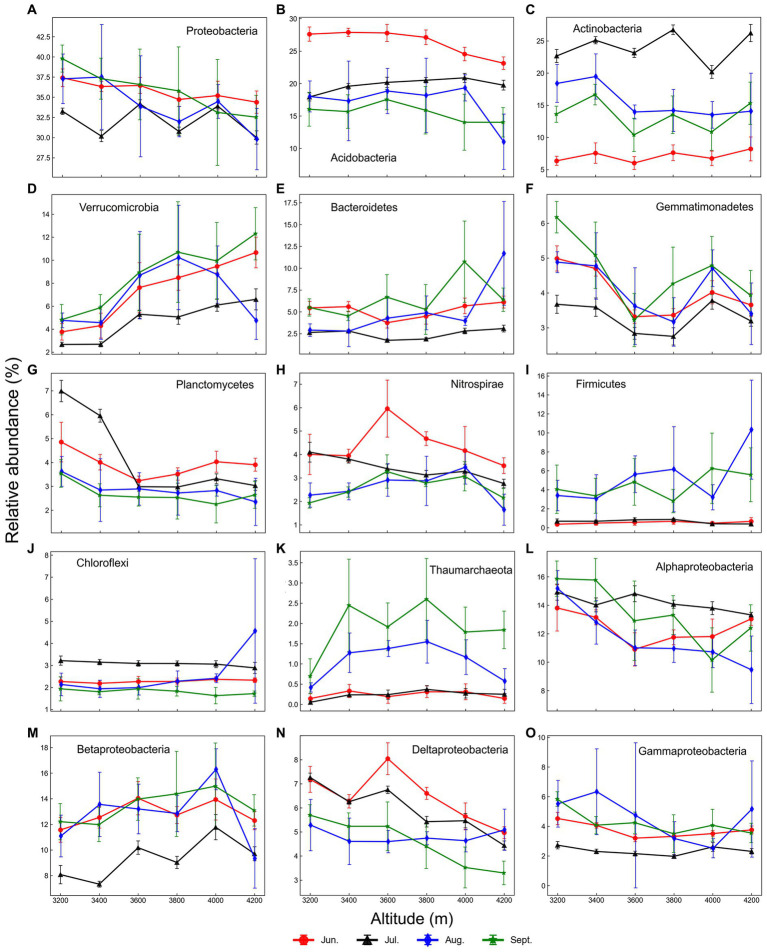
Elevational shifts of important phyla **(A–K)** and proteobacterial classes **(L–O)** in each month. Error bars represent standard deviations (*n* = 6).

Across all samples, 58 phyla and 943 genera were detected. Proteobacteria, Actinobacteria, and Acidobacteria were dominant phyla, accounting for >60% of relative abundance in most samples (Figure 3). Besides, Verrucomicrobia, Bacteroidetes, Gemmatimonadetes, Planctomycetes, Nitrospirae, Chloroflexi, Firmicutes, and Thaumarchaeota were also important phyla with high relative abundance.

Elevational patterns of community composition were diverse at the genus/phylum level, including increase (type I, genus level 2.3%/phylum level 4.9%), decrease (type II, 7.8%/3.3%), hump-shaped (type III, 3.2%/13.1%), U-shaped (type IV, 1.4%/1.6%) and no trend (type V, 85%/77%) (Supplementary Figures S5–S9). For example, Verrucomicrobia and Betaproteobacteria (mainly Nitrosomonadaceae) increased with elevation, while Alphaproteobacteria decreased (Figure 3). Patterns of Nitrospirae, Thaumarchaeota, and Deltaproteobacteria were hump-shaped, while Gemmatimonadetes was opposite. Besides, elevational patterns of many phyla were no trend, such as Acidobacteria, Actinobacteria, Firmicutes, and Chloroflexi. Besides, many rare taxa did not show any trends because of low abundance and low frequency.

At the genus level, type I genera were mainly composed of *Nitrospira*, *Rhodoplanes*, *Rhodomicrobium*, *Chthoniobacter*, *Bauldia*, and *Terrimicrobium* (Supplementary Figure S5). Several actinobacterial genera were type II, such as *Oryzihumus*, *Blastococcus*, *Mycobacterium*, and Arthrobacter (Supplementary Figure S6). Moreover, some rhizobia also belonged to type II, such as *Mesorhizobium*, *Rhizobium*, and *Devosia*. Type III genera were hump-shaped, such as *Nitrososphaera* and *Candidatus Solibacter* (Supplementary Figure S7). Type IV genera were lower at middle elevations, such as acidobacterial genera *Terrimonas* and *Aridibacter* (Supplementary Figure S8). Type I and II genera were significantly correlated to soil temperature, aboveground biomass, and altitude (Supplementary Table S1). Most type III and IV genera were significantly correlated to soil moisture.

### Seasonal dynamics of prokaryotic diversity and community composition

Species richness exhibited more pronounced seasonal changes than evenness (Supplementary Figure S10). Both richness and evenness were lowest in September. Richness indices declined from July to September (Supplementary Figures S10A,B). However, richness indices in June were not significantly different from those in July. Precipitation was important to seasonal dynamics of alpha diversity at 3200 m, negatively correlated to these indices, especially to richness (*p* < 0.01, Supplementary Table S2).

The community structures in June were quite different from those in other months, which was obvious along axis 2 of PCoA (Figure 2A). Moreover, samples in July were separated from those in August and September by axis 3 (Figure 2B). Community dissimilarities between sample groups (i.e., between elevations and months) were higher than those within each group (Supplementary Figure S2). Therefore, prokaryotic community succession during the growing season could be divided into 3 stages, that is early stage (June), middle stage (July), and late stage (August and September). Community structures (i.e., beta-diversity) were significantly different among months, even between the same month in different years (Figure 2D). According to Adonis’ analysis, altitude, sampling season and their interaction could explain 19%, 22%, and 12% of community variations in all samples, respectively, while 22%, 14%, and 12% if samples in June were excluded, indicating that the difference between early stage and other stages contributed more to community variation.

Seasonal dynamics of the prokaryotic community were mainly affected by aboveground biomass, precipitation, and temperature (Tables 1, 2, Figures 2C,D). Aboveground biomass was more important to seasonal dynamics at low elevations, since it increased from June to August (Supplementary Figure S1E), while temperature played more important roles at higher elevations (Tables 1–3). However, the seasonal changes of aboveground biomass were less significant at high elevations (Supplementary Figure S1E), resulting in less importance of aboveground biomass. The community dispersions were high at 3800 and 4,200 m (Supplementary Figure S4), weakening the impacts of temperature. Soil nitrate was significantly correlated to prokaryotic community structures at 3800 m (Tables 1, 2), indicating the importance of nitrogen cycling-related microorganisms there. Results at 3200 m revealed that precipitation was more important than soil moisture to seasonal dynamics (Tables 1–3; Figures 2C,D).

Seasonal patterns of community composition were also diverse during the growing season in 2021, including increase (type 1, 44%/36%), decrease (type 2, 9.1%/28%), hump-shaped (type 3, 8.6%/6.9%), V-shaped (type 4, 1.3%/3.4%), and no trend (type 5, 37%/26%) at the genus/phylum level. For example, Thaumarchaeota and Firmicutes increased and were most abundant at the late stage (i.e., August and September, type 1, Supplementary Figure S11), while Acidobacteria, Nitrospirae, and Deltaproteobacteria (mainly Syntrophobacteraceae) were most abundant in June and decreased during the growing season (type 2). Actinobacteria were most abundant in July (type 3), while Proteobacteria (such as beta and gamma classes), Gemmatimonadetes, and Verrucomicrobia indicated lowest in July (type 4). Those phyla with no trend were mainly rare taxa.

### Community assembly processes of abundant, intermediate, and rare species

**Figure 4:**
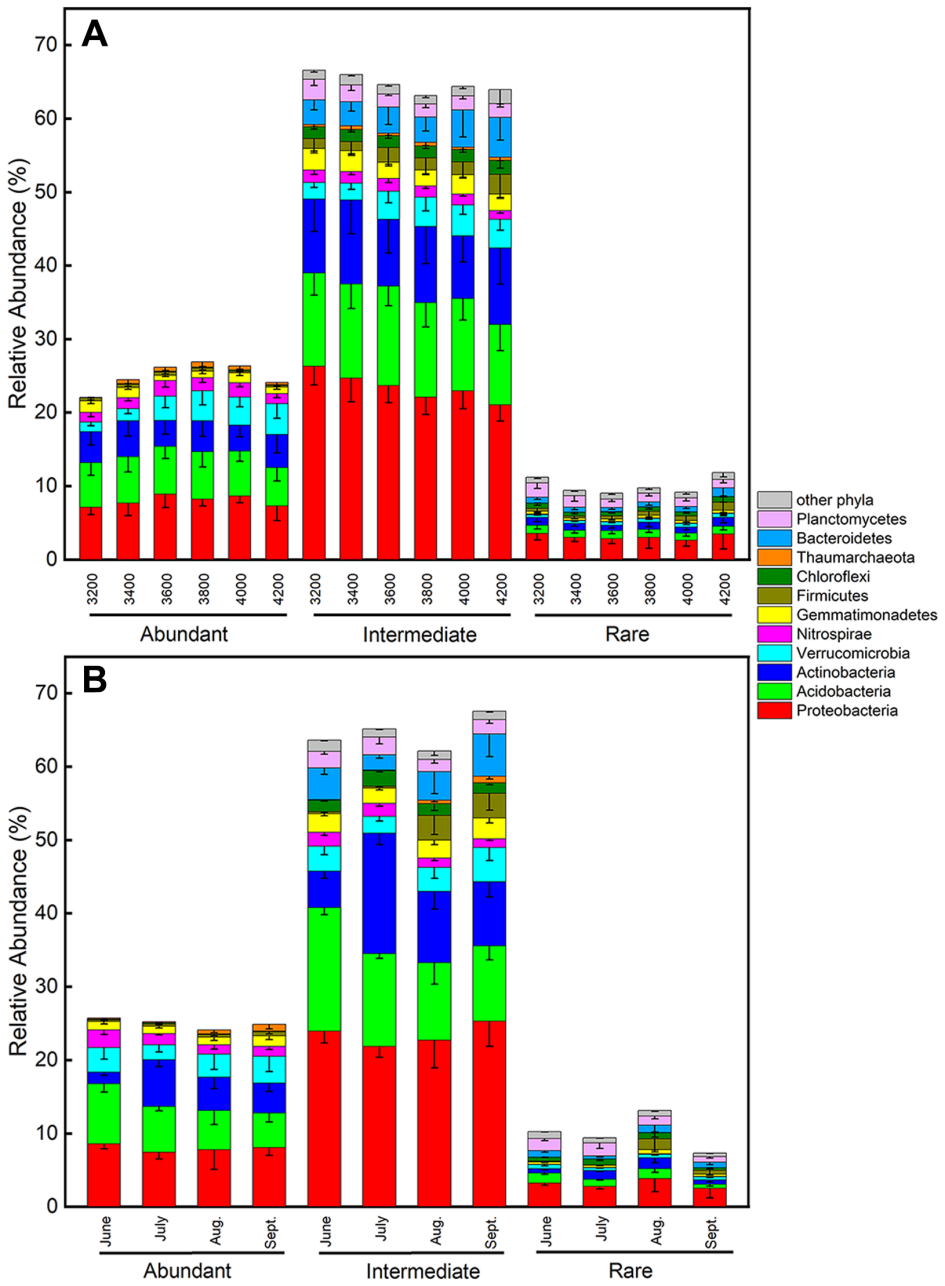
Relative abundances of phyla for abundant, intermediate and rare subcommunities **(A)** along the elevational gradient and **(B)** during the growing season. Error bars represent standard deviations (*n* = 6).

Across all samples, abundant, intermediate, and rare species consisted of 116, 10,568, and 31,065 ASVs, respectively, and their total relative abundances were 24.8%, 65.6%, and 9.6%, respectively (Figure 4). The PCoA plots of abundant and intermediate species were similar to those of all samples, that is, the subcommunity structure in June was quite different from other months (Supplementary Figures S12A,B). Key factors affecting these subcommunities were also similar to that of all samples, especially the intermediate species (Tables 1–3, Supplementary Table S3). However, the subcommunity structure of rare species in September was quite different from other months (Supplementary Figures S12C,D). The key factors influencing rare subcommunities were nitrate, C: N ratio, plant richness, and aboveground biomass (Supplementary Table S3).

**Figure 5:**
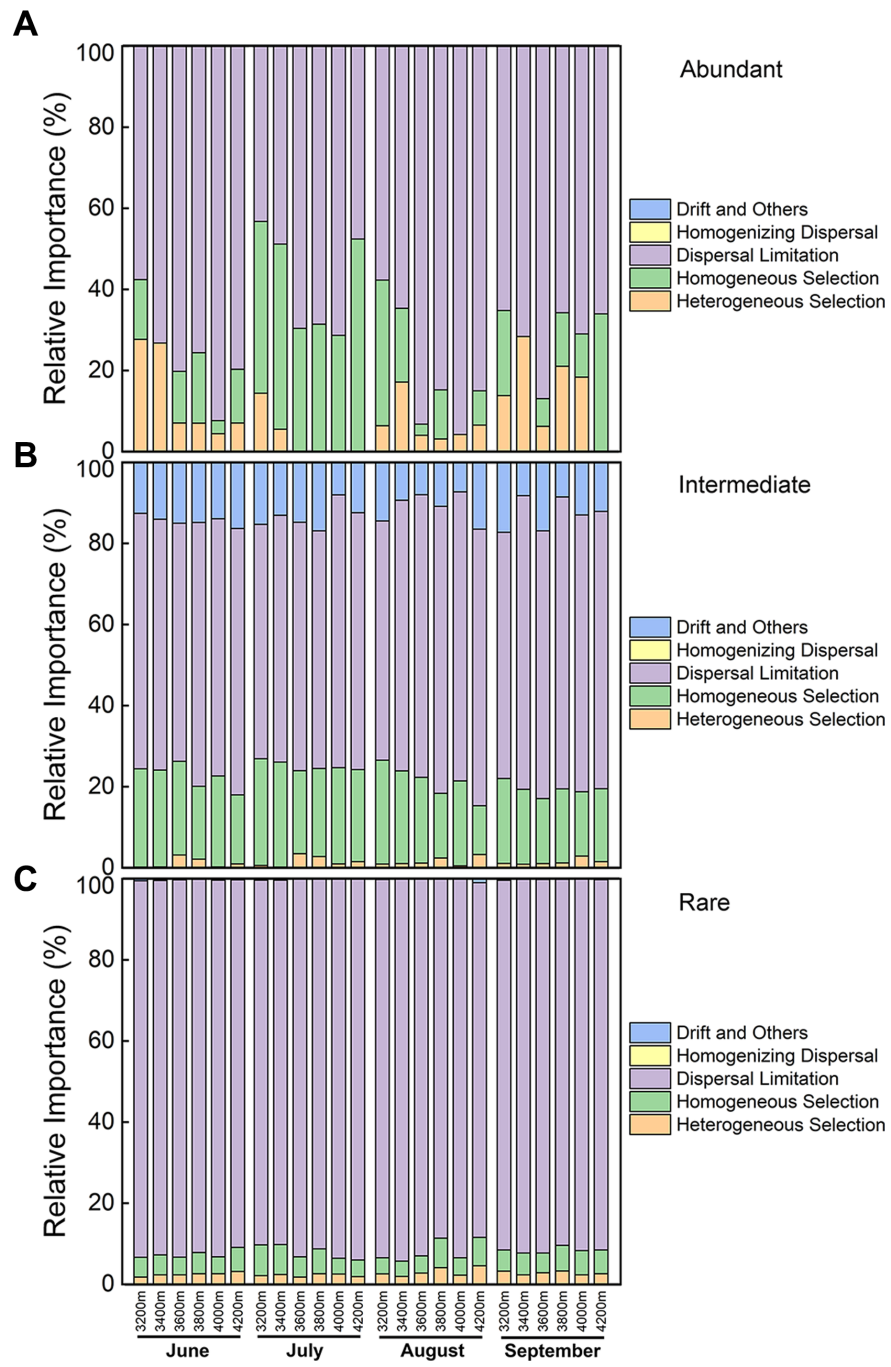
Relative importance of different ecological processes for **(A)** abundant, **(B)** intermediate, and **(C)** rare subcommunities along the elevational gradient during the growing season.

The primary assembly mechanism, particularly in rare subcommunities, was dispersal limiting (Figure 5). The proportions of contribution from different assembling processes were relatively steady in rare and intermediate subcommunities. The relative importance of homogeneous selection was higher in intermediate subcommunities than that in rare subcommunities. Besides, ecological drift was almost only observed in intermediate subcommunities.

In abundant subcommunities, the relative importance of different processes shifted variously, especially homogeneous selection and heterogeneous selection. The elevation pattern of the deterministic process was opposite to that of moisture, and this process was higher in drier July. Thus, water conditions (precipitation/moisture) might be the key factor affecting the balance between deterministic and stochastic processes in abundant subcommunities. Generally, dispersal limitation was lower at low elevations but higher at middle elevations, and the lowest in July (Figure 5A). Its relative importance was contributed by almost all abundant species (Supplementary Table S4). The homogeneous selection was higher in drier July, and it was also higher at 3200 and 4,200 m, indicating drought might increase its relative importance. Its relative importance was mainly contributed by Betaproteobacteria in June, Actinobacteria (“bin2”) in July, while Acidobacteria (classes Vicinamibacteria and Holophagae) and Verrucomicorbia in September (Supplementary Table S4). The heterogeneous selection was higher at low elevations, while it was lower at middle elevations, especially in July. Its relative importance was mainly contributed by Acidobacteria (classes Blastocatellia and Gp16) in August and September, while by Actinobacteria (“bin2”) in June (Supplementary Table S4). Interestingly, the roles of “bin2” changed, and its relative abundance increased dramatically from wetter June to drier July (Supplementary Figure S13). Therefore, community assemblies of different taxa might be different even though in the same place.

## Discussion

The main finding of this study was that soil prokaryotic diversity and community composition significantly shifted along the elevational gradient, mainly driven by soil temperature, followed by soil moisture. Seasonal patterns of community composition were also diverse and driven mainly by aboveground biomass, precipitation, and temperature. Water conditions might be the key drivers for the balance between deterministic and stochastic processes in abundant subcommunities along the elevational and seasonal gradients.

### Elevational shift of prokaryotic diversity and community composition

Our results clearly showed that temperature was the critical factor driving prokaryotic elevational distribution. Temperature is one of the most important factors affecting soil microbial community. First, high temperatures may directly alter the microbial community by increasing the metabolism and growth rates of microorganisms (Brown et al., 2004). Second, a higher temperature may increase aboveground biomass and alter plant community composition, then indirectly affecting the soil microbial community (Wang et al., 2009). Third, a higher temperature may increase the degradation rate of organic matter such as litter, and then increase the substrates for microbes (Zhou et al., 2016). In our study, aboveground biomass and richness were higher at low elevations with higher temperatures (Supplementary Figure S1). Our previous study showed that temperature indirectly affected prokaryotic communities through vegetation (Rui et al., 2015). Some important taxa were affected by temperature. For instance, Verrucomicrobia and Betaproteobacteria increased with elevation, while Alphaproteobacteria decreased (Figure 3). Verrucomicrobia is a common oligotroph in soils, adaptable to the high elevation where available nutrients are limited (Lipson and Schmidt, 2004; Yuan et al., 2021). Nitrosomonadaceae, the ammonia-oxidizing bacterium (AOB) (Garrity et al., 2015), was the most abundant family in Betaproteobacteria in this study, which increased with elevation. Many members of Alphaproteobacteria are copiotrophs, resulting in the decrease pattern of this class, in line with our previous study (Rui et al., 2015). Moreover, alphaproteobacterial genera *Mesorhizobium*, *Rhizobium*, and *Devosia* declined with elevation, which are symbiotic rhizobia associated with legumes (Franche et al., 2009). Our previous study showed that legume biomass is higher at low elevation, resulting in higher symbiotic nitrogen fixation and a higher abundance of symbiotic rhizobia (Rui et al., 2022).

Soil moisture was another important factor driving prokaryotic elevation distribution, although its effect was smaller than temperature (Tables 1–3). The physiological state of microorganisms could be directly influenced by soil moisture (Prado and Airoldi, 1999), and indirectly affect them by altering vegetation and soil properties (McHugh et al., 2014; Chen et al., 2015). Excessive soil moisture may lead to an anaerobic environment unfavorable to aerobic microbes (Unger et al., 2009). Due to the influence of topography on rainfall in this study, the soil moisture got maximum at 3600 m during the growth season at the study location (Xu et al., 2010). Many soil properties were significantly correlated to moisture, such as TN, TOC, nitrite contents, and pH (*p* < 0.001). Our previous study showed that anaerobic free-living diazotrophs are most abundant at 3600 m, indicating the anaerobic microenvironment there (Rui et al., 2022). In this study, some type III taxa (hump-shaped) were anaerobic or facultatively anaerobic, such as *Defluviicoccus*, *Hyphomicrobium,* and *Phaselicystis* (Supplementary Figure S7). *Nitrososphaera*, known as common ammonia-oxidizing archaea (AOA) in soils (Tourna et al., 2011), was higher at mid-elevation. The positive correlation between *Nitrososphaera* and soil moisture/precipitation is identical to other studies (Gu et al., 2017; Yang et al., 2018). In contrast, type IV phylum Gemmatimonadetes was U-shaped. Gemmatimonadetes prefers low moisture (DeBruyn et al., 2011), and is abundant in dry soils (Singh et al., 2014). Moisture also indirectly affected microbial taxa through soil pH. For example, *Candidatus Solibacter*, a member of Acidobacteria subdivision 3, was hump-shaped and displayed highest at 3600 m because low pH was preferred (Halamka et al., 2022). The U-shaped *Aridibacter* and *Blastocatella* are members of Acidobacteria subdivision 4, which prefers high pH, common in arid soils and crusts (Foesel et al., 2013).

Soil pH is often recognized as the critical driver of microbial elevational distribution in the literature (Bryant et al., 2008; Looby and Martin, 2020). However, it is not the case in this work. The importance of soil pH depends on the range of soil pH. A very narrow range of pH perhaps lead to less importance of soil pH (Singh et al., 2014). Interestingly, substantial changes in soil pH are often observed in elevational gradients where soil pH is the key factor driving bacterial elevational distribution (Shen et al., 2013; Xu et al., 2014; Li et al., 2018). In our study, the range of soil pH was narrow (Supplementary Figure S1), resulting in less importance of soil pH.

In summary, soil temperature and moisture drove the elevational distribution of soil prokaryotic community. Copiotrophs and symbiotic diazotrophs declined with elevation, while oligotrophs and AOB increased, affected by temperature. Anaerobic or facultatively anaerobic bacteria and AOA were hump-shaped, mainly influenced by moisture.

### Seasonal dynamics of prokaryotic diversity and community composition

In this study, we found obvious seasonal dynamics of soil prokaryotic community at each elevation, which were mainly affected by aboveground biomass, precipitation, and soil temperature. Plants primarily influenced the soil microbial community by adding litter and root exudates (Knelman et al., 2012). Root exudates may change during the plant growth cycle, and alter soil microbial community structures, especially in rhizospheres (Dunfield and Germida, 2003; Li et al., 2014). At the early stage of the growing season, aboveground biomass was much lower than in the mid and late stages, indicating available carbon input from plants might be also lower. In this study, relative abundances of Acidobacteria, Nitrospirae, and Deltaproteobacteria were relatively higher in June (Supplementary Figure S11). Acidobacteria is a keystone oligotroph in soils, abundant in bulk soils with low resource availability (Fierer et al., 2007). It plays an important role in the degradation of soil organic matter (Banerjee et al., 2016). *Nitrospira*, the dominant genus of Nitrospirae in our study, is able to oxidize ammonia to nitrite (Daims and Wagner, 2018), perhaps related to high ammonium and low nitrite contents in June (Supplementary Figure S1). Deltaproteobacteria were dominated by Syntrophobacteraceae, related to anaerobic degradation of organic substrates and sulfate reduction (Kuever et al., 2015). Interestingly, relative abundances of rhizobia, such as *Bradyrhizobium*, *Mesorhizobium*, and *Rhizobium*, were the lowest in June, owing to the low biomass of legumes. Thus, symbiotic nitrogen fixation might be less important in June, and the high ammonium content could be from other pathways, such as free-living nitrogen fixation (Rui et al., 2022). It is necessary to investigate the seasonal dynamics of the diazotrophic community and measure the nitrogen fixation rate here in the future.

Actinobacteria plays important roles in carbon cycling, especially in the solubilization of fungal and plant cell walls (Lacombe-Harvey et al., 2018). The relative abundance of Actinobacteria was the lowest in June due to the lowest aboveground biomass. When aboveground biomass increased with lower precipitation in July, Actinobacteria increased dramatically and reached the highest level (Supplementary Figure S11). The relative abundance of Actinobacteria was negatively correlated to precipitation in soils during July and September (*p* < 0.001), indicating precipitation became the main factor for Actinobacteria, in line with another study (Yao et al., 2017). Seasonal dynamics of Actinobacteria in grassland soils were also observed in another study, caused by seasonal variation of rainfall (Jenkins et al., 2009). In our study, several actinobacterial genera such as *Arthrobacter* were positively correlated to aboveground biomass, which prefer soils receiving organic inputs (Jenkins et al., 2009). Compared to other phyla, the relative abundance of Actinobacteria changed dramatically during the growing season. Thus, Actinobacteria was the keystone indicator of seasonal dynamics of the soil prokaryotic community in the alpine meadow.

Our study showed that several important phyla changed almost linearly during the growing season. Community structures were more similar between the same months in different years than those between different stages (Figure 2). The seasonal dynamics indicated that the soil prokaryotic community was sensitive to short-term climate change, such as monthly variation of precipitation. Thus, seasonal variation should be paid attention to the time of soil sampling for bacterial community sequencing.

### Community assembly processes of abundant, intermediate, and rare species

We found that dispersal limitation was the main assembly process for the prokaryotic community in alpine grassland soils, in accordance with the transect study across the Qinghai-Tibetan Plateau (Kang et al., 2022). Two reasons could explain it. First, soil microbes produce dormancy to deal with stressors such as low temperature, and microbial dormancy serves as a seed bank that may weaken the selection effects of community assembly, but increase the relative importance of stochastic processes (Nemergut et al., 2013). Second, soil freezing is a physical barrier that can limit the movement of microbes (Steven et al., 2008), increasing dispersal limitation (Kang et al., 2022). Permafrost and seasonally frozen zones cover 96% of the areas on the Qinghai-Tibetan Plateau (Zou et al., 2017). Therefore, it is not surprising that dispersal limitation is the primary process for the prokaryotic community in alpine grassland soils (Kang et al., 2022).

Different ecological processes were dominant in different subcommunities. First, the relative importance of dispersal limitation was the highest in rare subcommunities. We suspect that the reason is that species with higher abundance may have a greater probability of dispersal than rare species (Nemergut et al., 2011; Zhou and Ning, 2017). In this study, abundant species were found in most samples, while rare species were in only 10 samples on average. Second, the relative importance of drift was the highest in intermediate subcommunities. Drift refers to random changes due to birth, death, and reproduction (Zhou and Ning, 2017). A large fraction of a rare biosphere consists of nongrowing cells, such as dormancy cells and spores (Pedrós-Alió, 2012). Therefore, drift was seldom observed in rare subcommunities in our study. Third, heterogeneous selection was more important to abundant subcommunities, while the homogeneous selection was more important to intermediate subcommunities. Heterogeneous selection (i.e., variable selection) refers to the heterogeneous changes of communities in selective pressure from environmental heterogeneous, while homogeneous selection refers to the homogeneous changes in a consistent selective pressure if environmental conditions are homogeneous (Dini-Andreote et al., 2015; Stegen et al., 2015). The environmental conditions were relatively homogeneous at the same elevation and at the same time, causing strong homogeneous selection. Abundant species usually grow fast and contribute more to nutrient cycling and carbon flow (Pedrós-Alió, 2012). Thus, the response of abundant species to selective pressure might be more active than immediate and rare species.

A transect research reported that precipitation can balance deterministic and stochastic processes in grassland soils (Yang et al., 2022). We found that water conditions might be the key factor affecting the balance between deterministic and stochastic processes in abundant subcommunities. Actinobacteria played an important role here (Supplementary Table S4). From wetter June to drier July, the relative abundance of Actinobacteria increased dramatically. As a result, communities at the same elevation became similar (Supplementary Figure S4), in line with the definition of homogeneous selection. Therefore, water conditions might be the critical factor influencing community assembly.

## Conclusion

In conclusion, soil prokaryotic diversity and community composition significantly shifted along the elevational gradient, mainly driven by soil temperature, followed by soil moisture. Elevational patterns of community composition were diverse, including increase, decrease, hump-shaped, U-shaped, and no trend. Moreover, seasonal patterns of prokaryotic community composition were also diverse and driven mainly by aboveground biomass, precipitation, and temperature. Actinobacteria was the keystone indicator of seasonal dynamics, more abundant in drier July. Dispersal limitation consistently dominated the assembly process of soil prokaryotic communities along both elevational and seasonal gradients. The deterministic process of abundant species was relatively higher at drier sites and in drier July. Our findings suggest that rapid changes in growing season climate can alter the patterns of soil prokaryotic biodiversity along both elevational and seasonal environmental gradients, especially carbon/nitrogen cycling microorganisms. This work highlights that temperature, water conditions, and plant productivity are critical factors driving soil prokaryotic community along elevations during the growing season, and provides new insights into the response of soil prokaryotic community to changes in elevation and season, and helps us understand the temporal and spatial fluctuations of the climate-sensitive Qinghai-Tibetan Plateau.

## Data availability statement

The datasets presented in this study can be found in online repositories. The names of the repository/repositories and accession number(s) can be found at: https://ngdc.cncb.ac.cn/gsa/browse/CRA009192, CRA009192.

## Author contributions

JR: Conceptualization, Data curation, Formal analysis, Funding acquisition, Methodology, Project administration, Software, Validation, Visualization, Writing – original draft, review & editing. YZ: Investigation, Resources, Writing – original draft. NC: Methodology, Writing – review & editing. FW: Investigation, Writing – original draft. CL: Investigation, Writing – original draft. XL: Resources, Writing – review & editing. JH: Investigation, Writing – original draft. NL: Funding acquisition, Writing – review & editing. XJ: Project administration, Supervision, Writing – original draft, review & editing.

## Footnotes

^1^ http://earthmicrobiome.org/protocols-and-standards/

^2^ http://www.drive5.com/usearch
