# Supplementary material for: Elevational distribution and seasonal dynamics of alpine soil prokaryotic communities
Source: Front Microbiol. 2023 Sep 22;14:1280011. doi: 10.3389/fmicb.2023.1280011 (PMC10557256; doi:10.3389/fmicb.2023.1280011)
Supplement: Supplementary file 1 [file Data_Sheet_1.docx]

Supplementary Material

Elevational distribution and seasonal dynamics of alpine soil prokaryotic communities

Junpeng Rui, Yuwei Zhao, Nan Cong, Fuxin Wang, Chao Li, Xiang Liu, Jingjing Hu, Ning Ling, Xin Jing *

*** Correspondence:** Xin Jing: [jingx@lzu.edu.cn](mailto:jingx@lzu.edu.cn)

# Supplementary Data

**Soil and plant properties**

During the growing season, soil temperature decreased linearly with elevation, and higher in July and August than that in June and September (Fig. S1 A). Elevational changes of aboveground biomass and plant richness were similar to soil temperature, but aboveground biomass at 4200 m was higher than that at 3800-4000 m, especially in July and August. Plant richness didn’t change significantly during the growing season, while aboveground biomass in June was the lowest, and increased till August (Fig. S1 DE). Based on 16S rRNA gene copies, soil prokaryotic biomass was higher at 3200-3600 m than 3800-4200 m. It was the lowest in June, and highest in July (Fig. S1 F).

Soil moisture increased linearly from 3200 m to 3600 m, and decreased at higher elevations. It was lowest in June and didn’t shift significantly during July and September (Fig. S1 B). Elevational changes of soil TN, TOC and nitrite contents were similar to moisture, but they were higher at 4200 m than those at 4000 m (Fig. S1 GHL), perhaps due to higher aboveground biomass there. Soil ammonium content decreased linearly from 3200 m to 3600 m, but didn’t change significantly at higher elevations. It declined from June to August and picked up in September (Fig. S1 J). Nitrate was higher at 3600-3800 m, especially in July and August (Fig. S1 K).

**Usage of Perl scripts**

All Perl scripts mentioned in this article are in Data Sheet 2 of the Supplementary Material. They are also available on GitHub (<https://github.com/PeterRui/perl4amplicon>). Alternatively, you can also obtain the latest versions from the scripts’ designer, Dr. Junpeng Rui ([peter_rjp@163.com](mailto:peter_rjp@163.com)). Please use them within command-line tools such as CMD in Windows, or Terminal in Linux.

(1) trim_primer_in_fq.pl

This script can remove primers and barcodes from fastq files. For example:

perl trim_primer_in_fq.pl -i “fq/*.fastq” -l primers.txt -d out -c 1

-i “fq/*.fastq”: all fastq files in the input directory “fq”

-l primers.txt: the text file including primers

-d out: the output directory

Note: Reads with more than one pair of primers will also be removed if you use -c 1.

(2) subsample_in_table.pl

This script can normalize the number of sequences in an ASV/OTU-table. In other words, the total numbers of reads in all samples will be the same after random subsample. The input file is an ASV/OTU-table. For example:

perl subsample_in_table.pl -i [input file] -o [output file] -s [number of samples]

(3) alpha_diversity.pl

This script can calculate alpha diversity indices such as Chao1 richness, Observed species, Shannon diversity, Simpson index, Pielou evenness, and Good’s coverage. The input file is an ASV/OTU-table. For example:

perl alpha_diversity.pl -i [input file] -o alpha_div.txt -s [number of samples]

(4) percent_in_table.pl

This script can convert sequence numbers into relative abundance (%) in an ASV/OTU-table. The input file is an ASV/OTU-table. For example:

perl percent_in_table.pl -i [input file] -n [number of samples]

(5) sum_taxa_from_otu_table.pl

This script can calculate the total abundance of each taxon at each taxonomic rank based on an ASV/OTU-table. The input file is an ASV/OTU-table. For example:

perl sum_taxa_from_otu_table.pl -i [input file] -s [number of samples] -D [data type]

-D: data type of the input file (default 0). 0 means relative abundance (%, i.e., percentage, the output file of percent_in_table.pl), while 1 means sequence numbers.

# Supplementary Figures and Tables

## Supplementary Figures


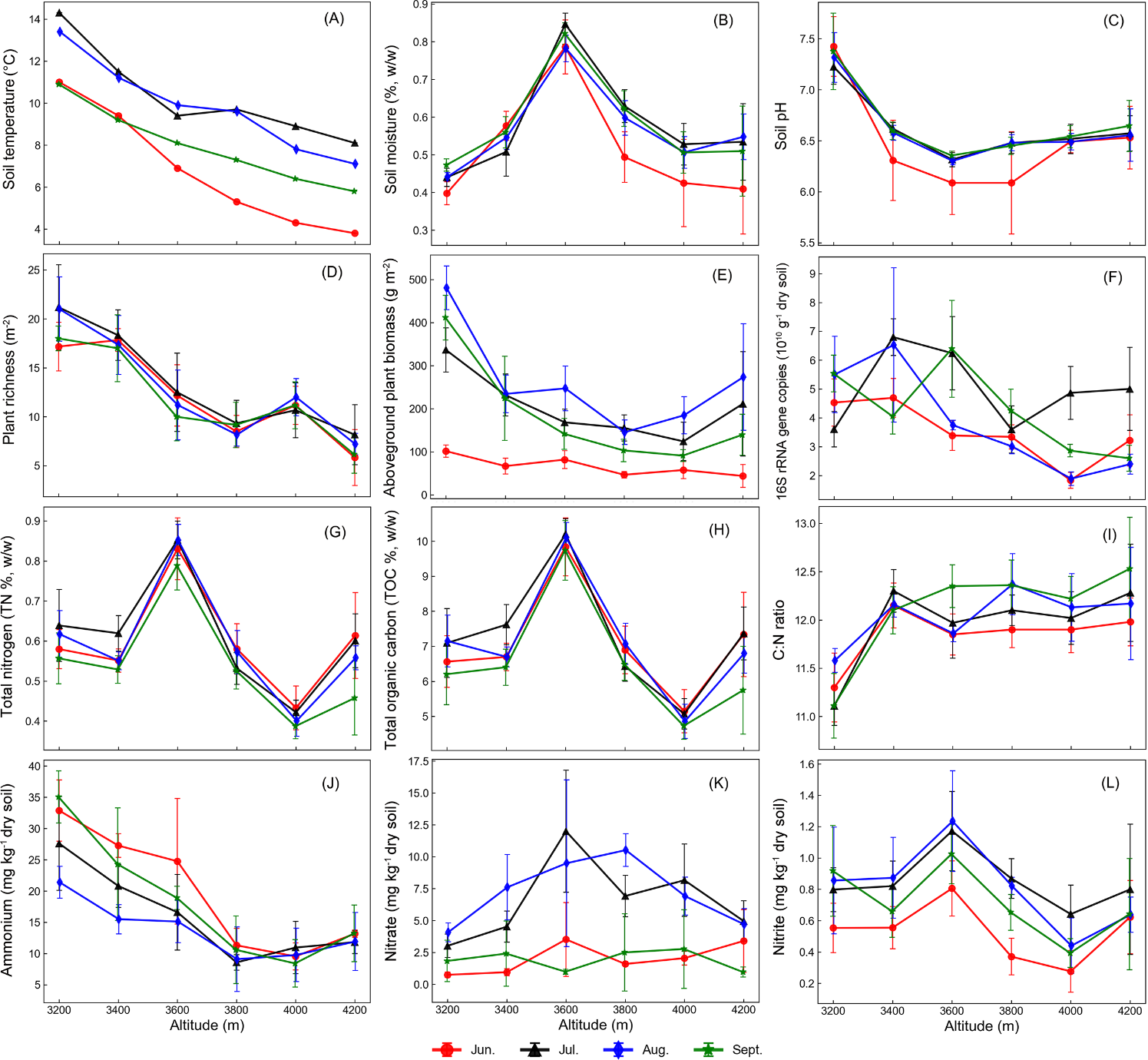


**Supplementary Figure S1.** Elevational changes of environmental factors during the growing season. Error bars represent standard deviations (n = 6).


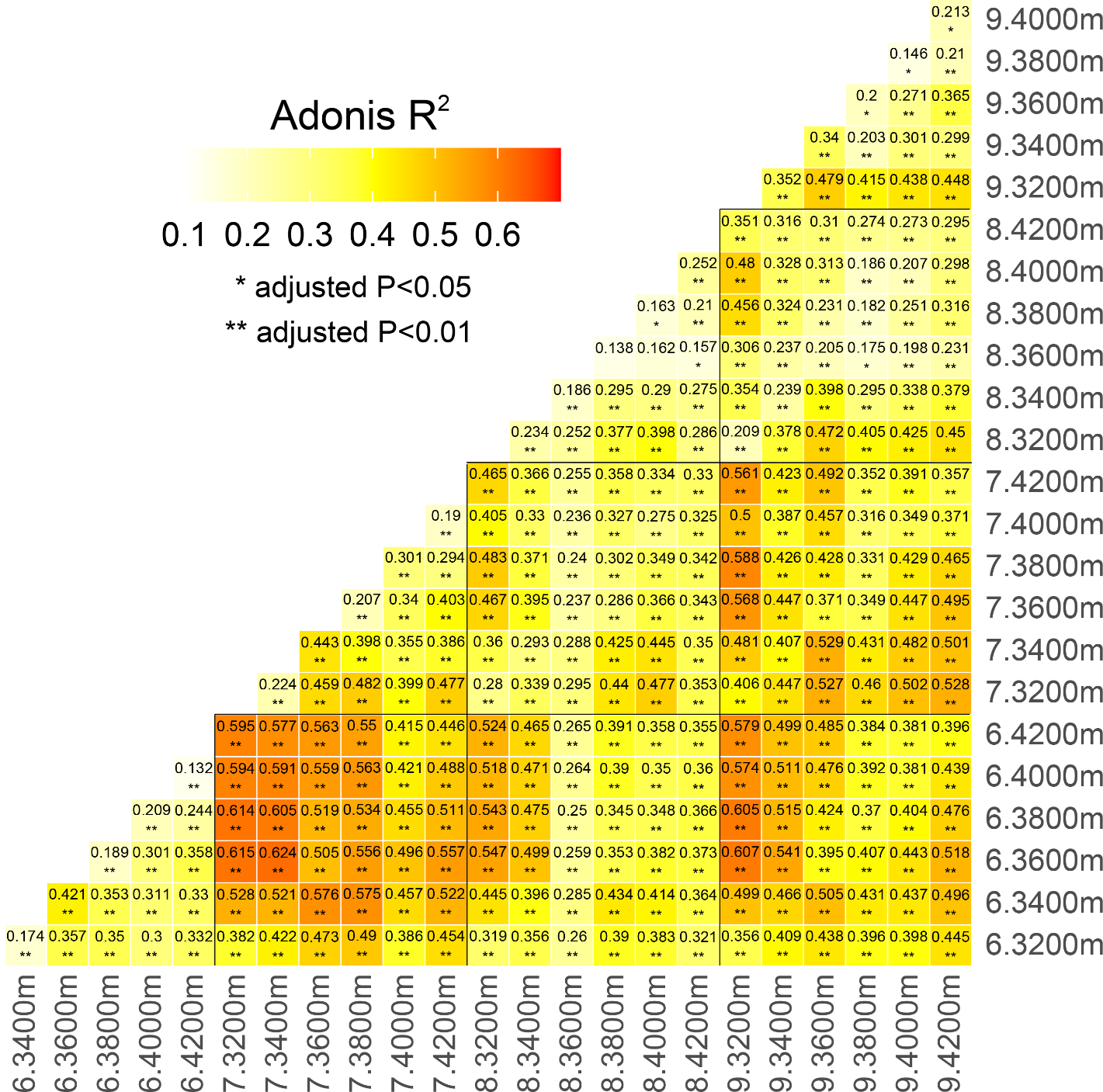


**Supplementary Figure S2.** R^2^ of pairwise Adonis between sample groups. “6.3200m” means samples at 3200 m in June.


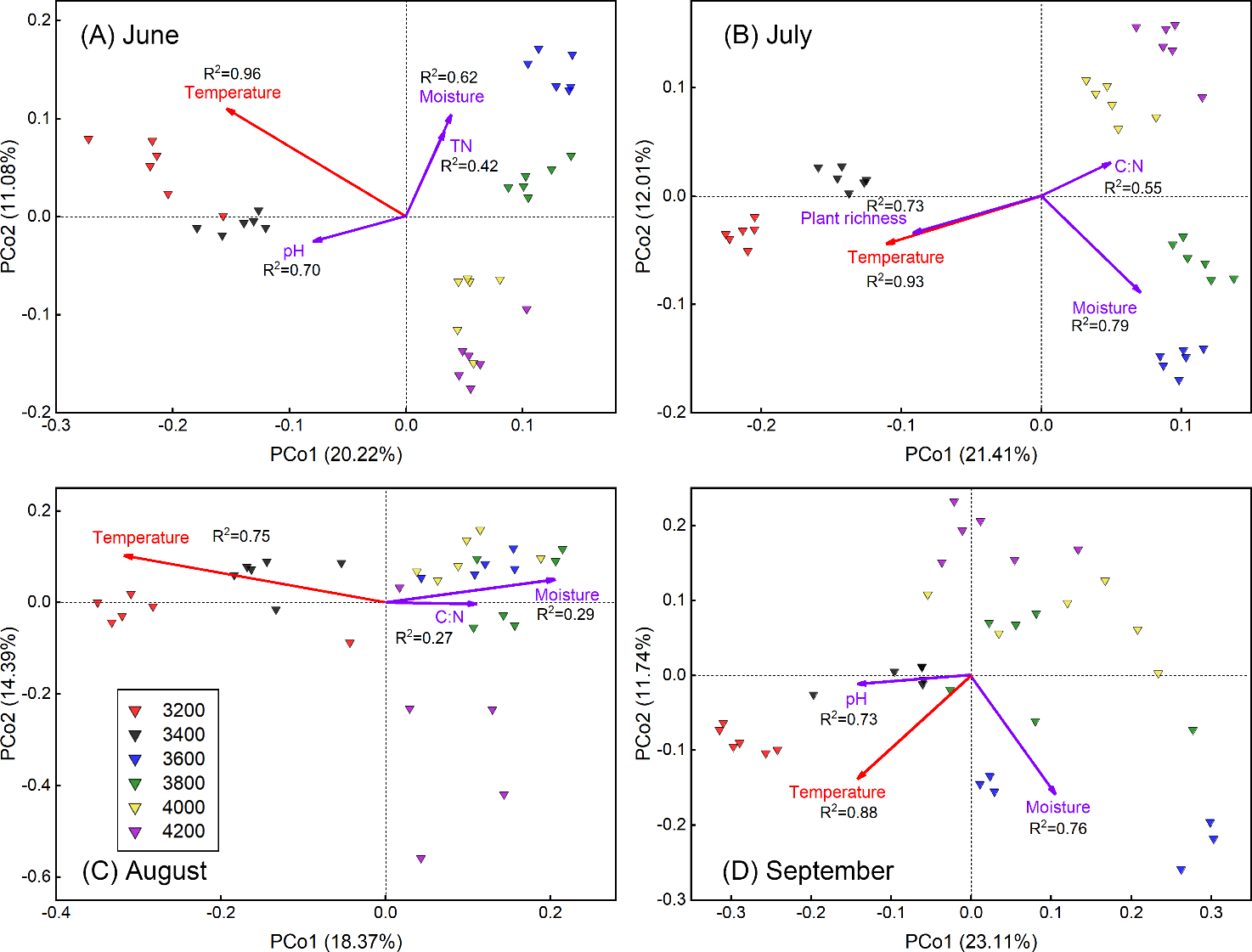


**Supplementary Figure S3.** PCoA plots of prokaryotic communities in each month. Important environmental factors were fitted onto the patterns. R^2^ values of fitness were labeled near the factor arrows. Values on axes indicated the percentages of total variation explained by each axis.


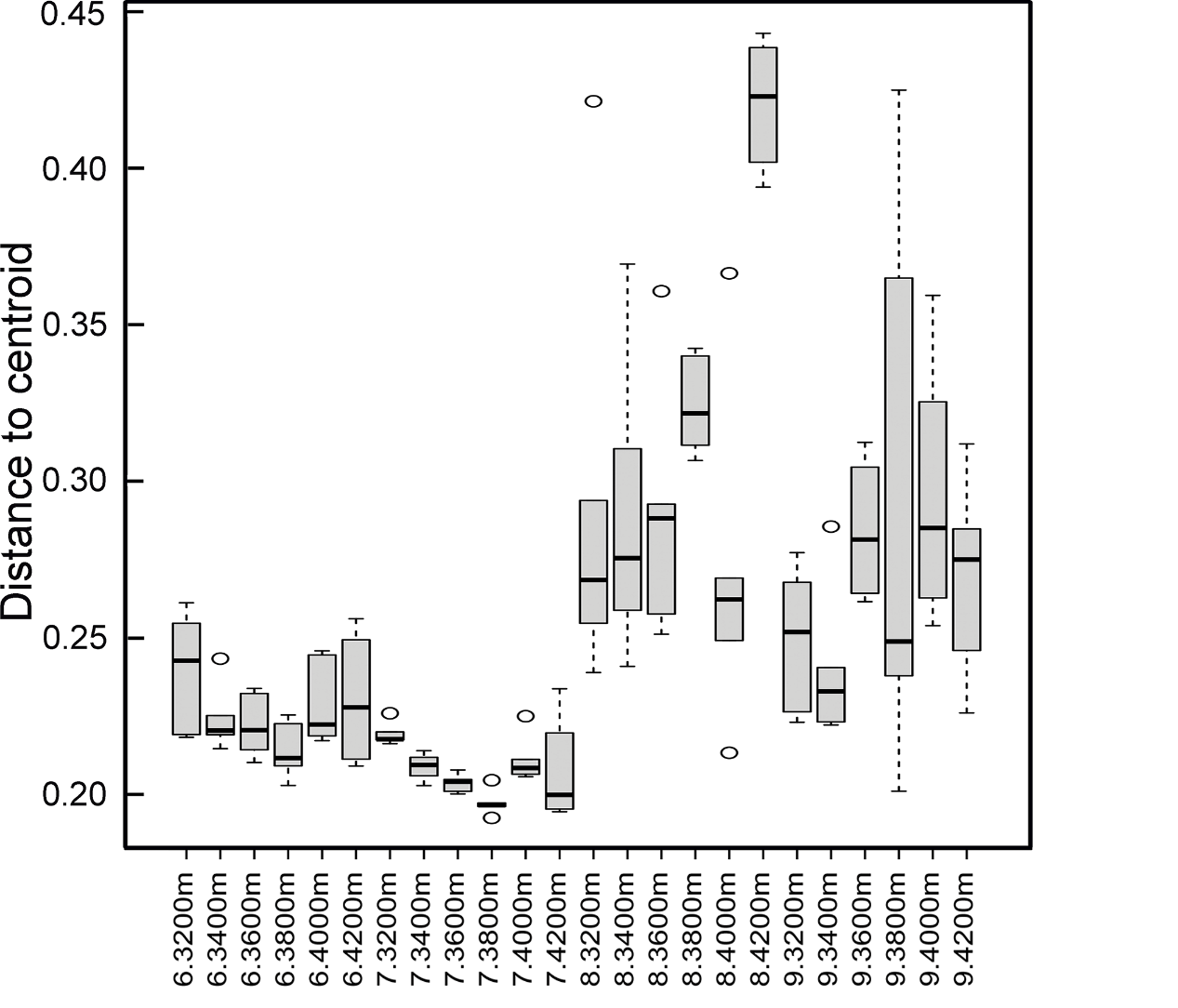


**Supplementary Figure S4.** Community distance to the centroid of each sample group based on PERMDISP. “6.3200m” means samples at 3200 m in June.


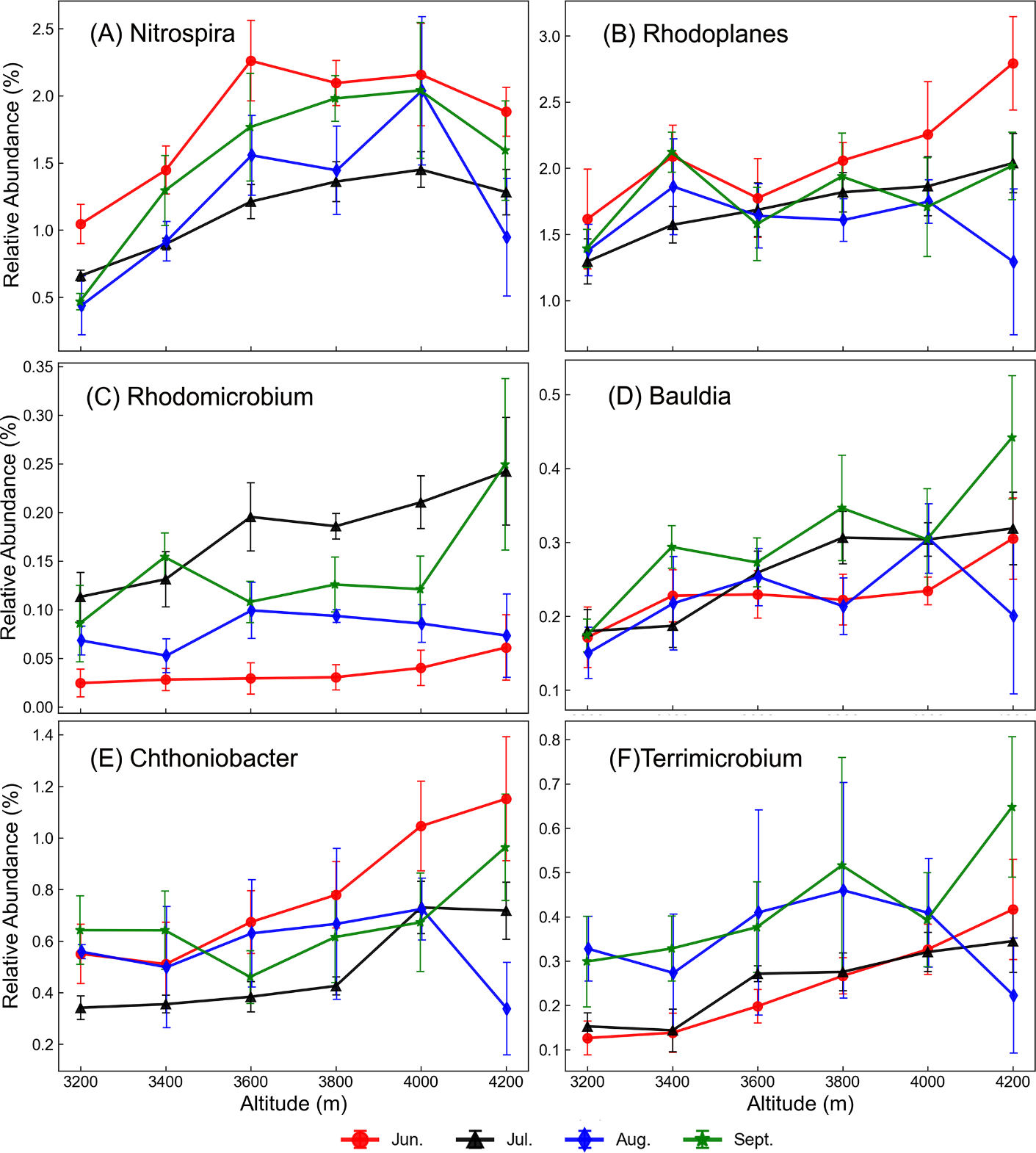


**Supplementary Figure S5.** Relative abundances of representative genera increasing with elevation (type I). Error bars represent standard deviations (n = 6).


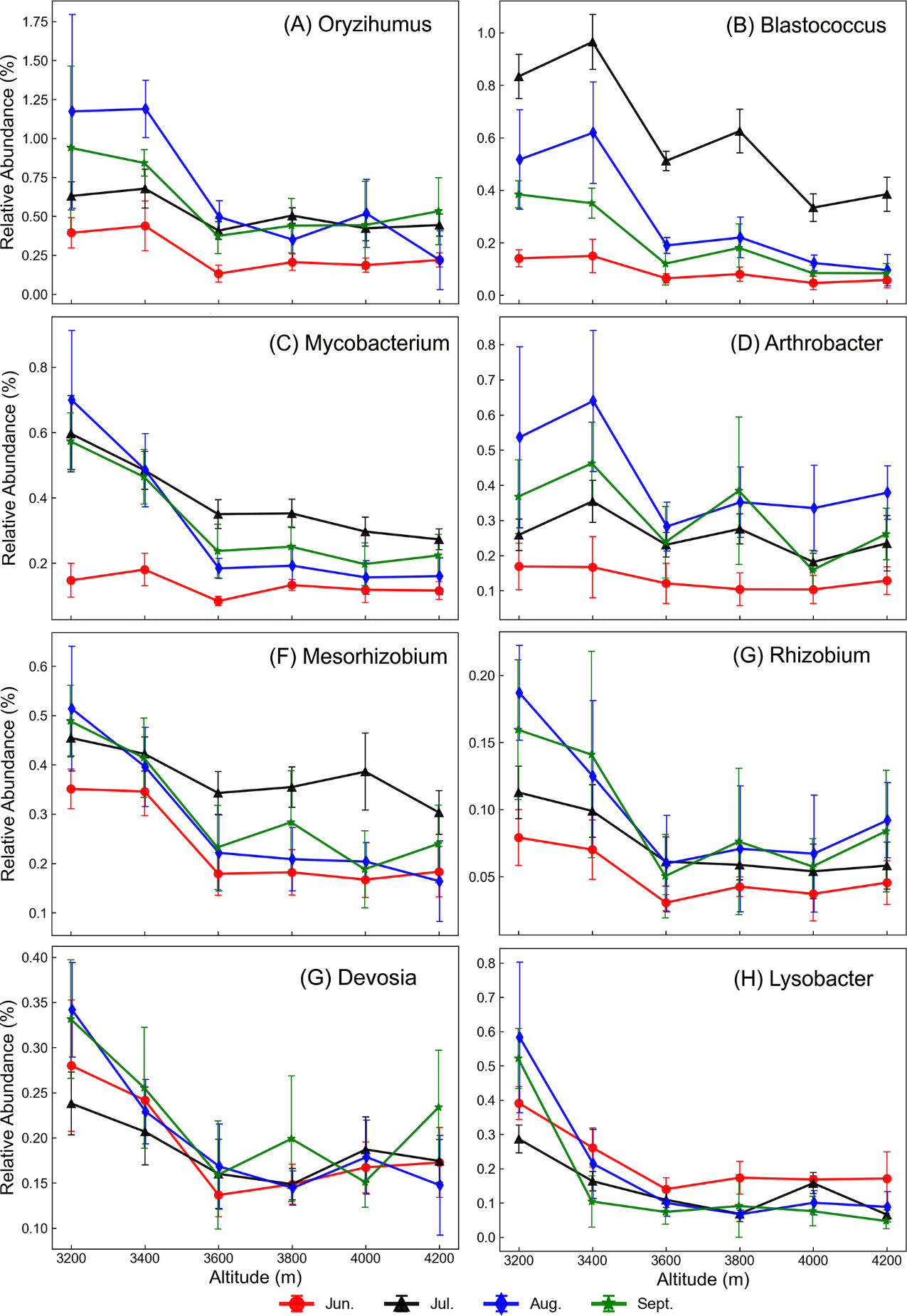


**Supplementary Figure S6.** Relative abundances of representative genera decreasing with elevation (type II). Error bars represent standard deviations (n = 6).


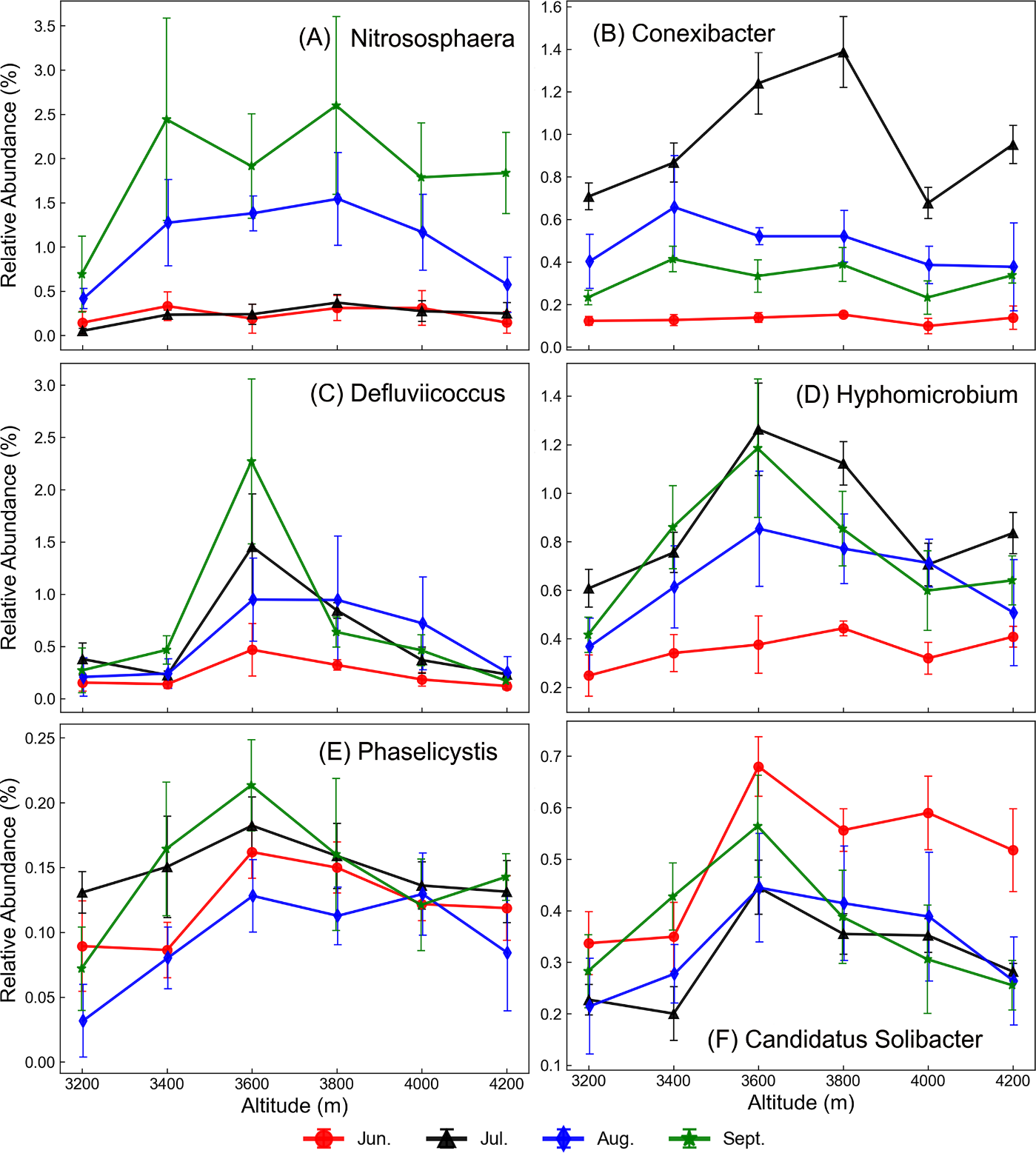


**Supplementary Figure S7.** Relative abundances of representative genera more abundant at mid-elevation (type III). Error bars represent standard deviations (n = 6).


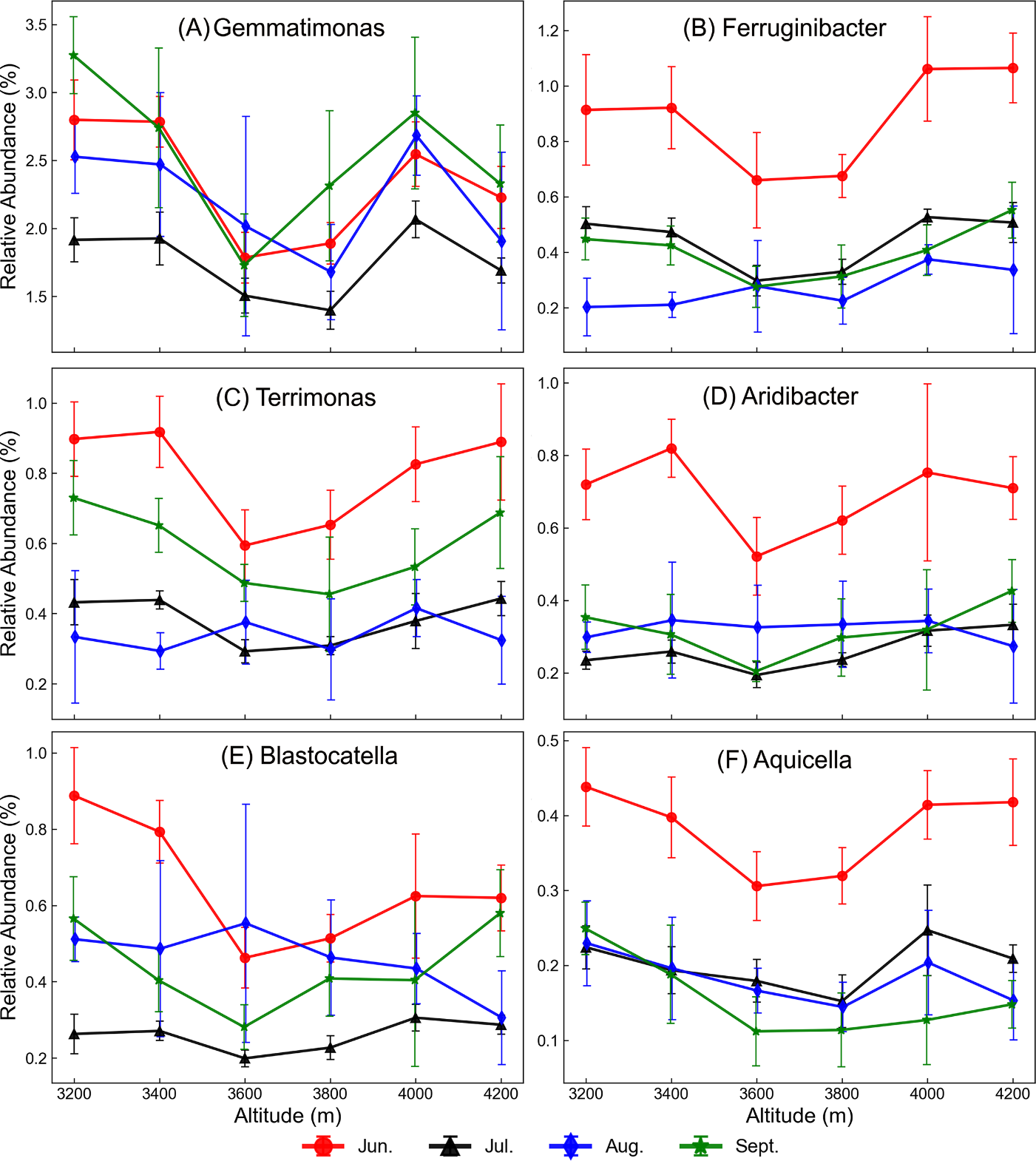


**Supplementary Figure S8.** Relative abundances of representative genera less abundant at mid-elevation (type IV). Error bars represent standard deviations (n = 6).


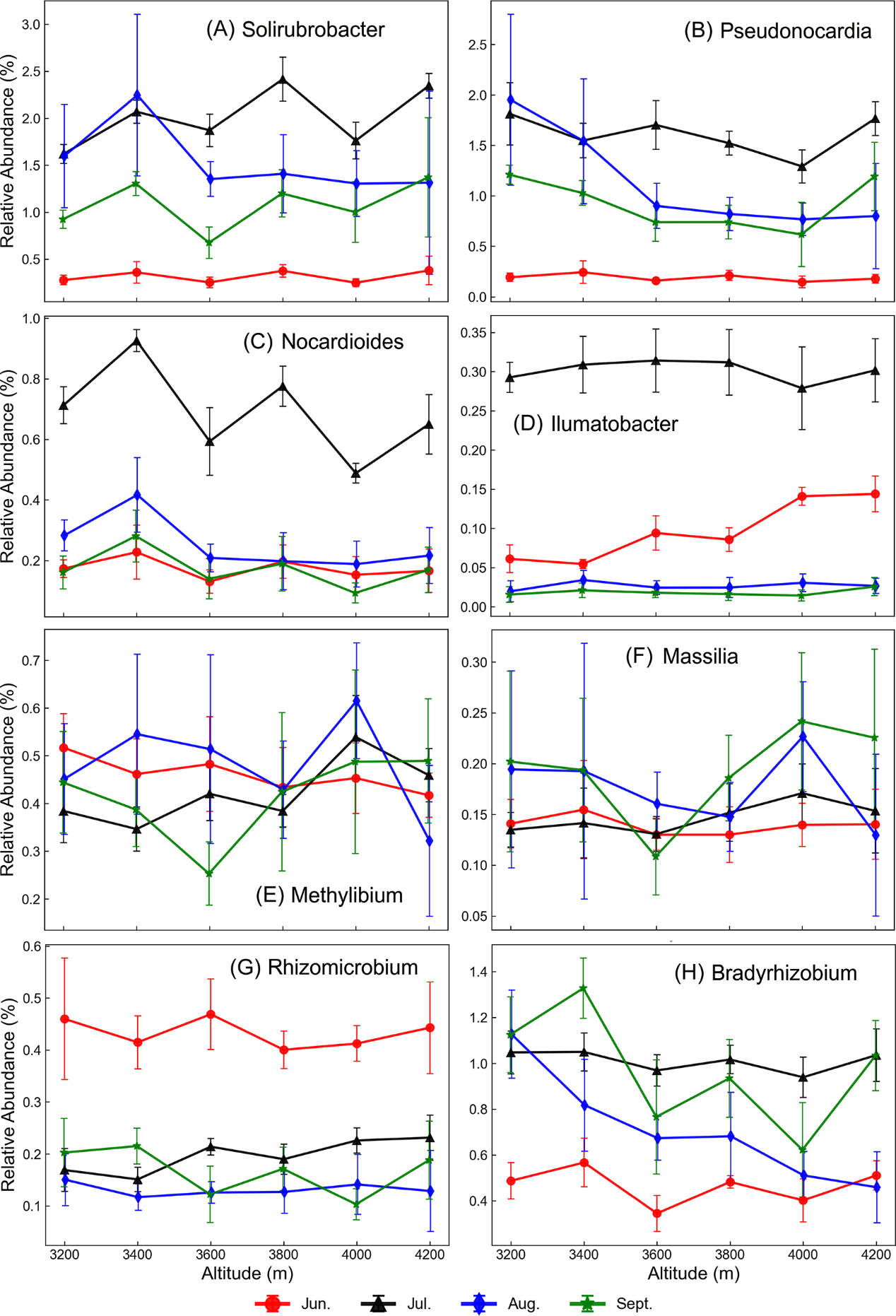


**Supplementary Figure S9.** Relative abundances of representative genera with not trend along elevation gradient (type V). Error bars represent standard deviations (n = 6).


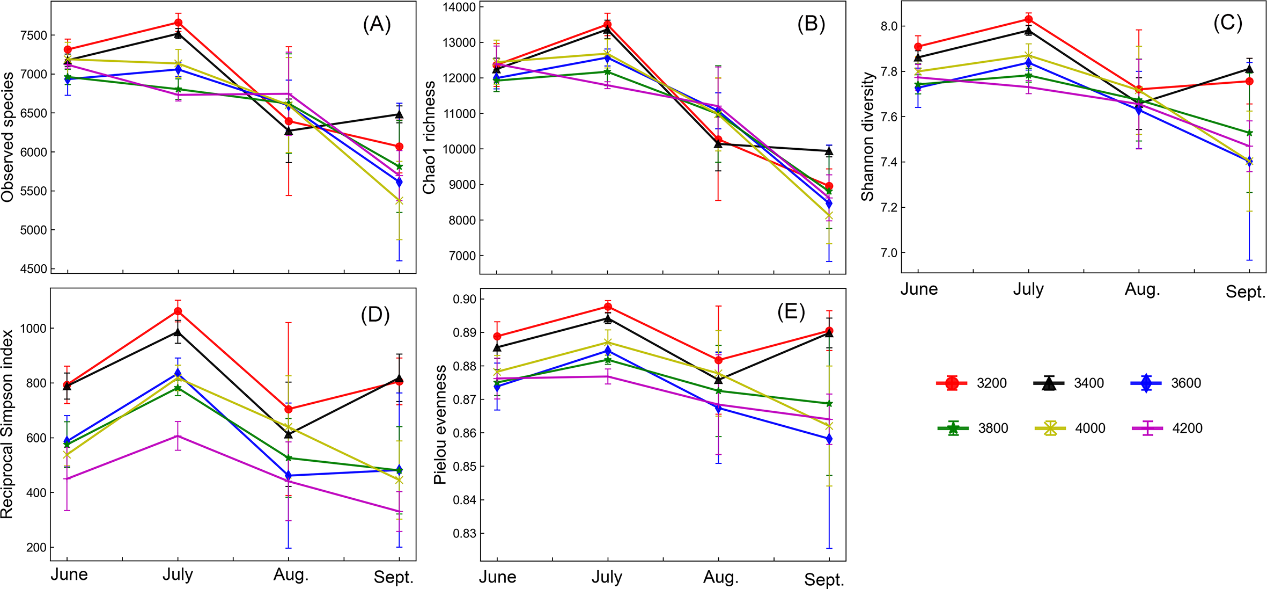


**Supplementary Figure S10.** Seasonal dynamics of prokaryotic alpha diversity indices at each elevation. Error bars represent standard deviations (n = 6).


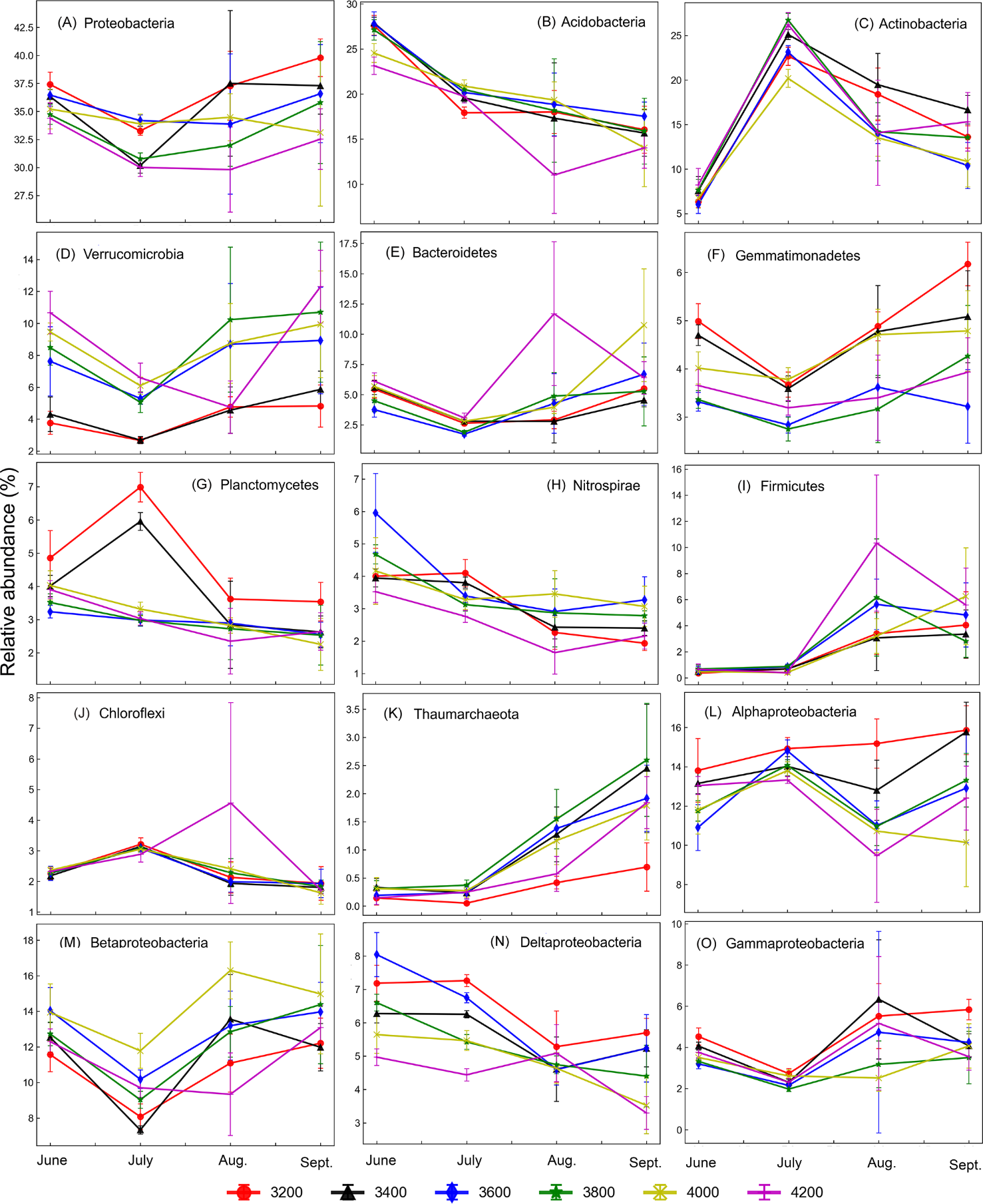


**Supplementary Figure S11.** Seasonal dynamics of important phyla (A-K) and proteobacterial classes (L-O) at each elevation. Error bars represent standard deviations (n = 6).


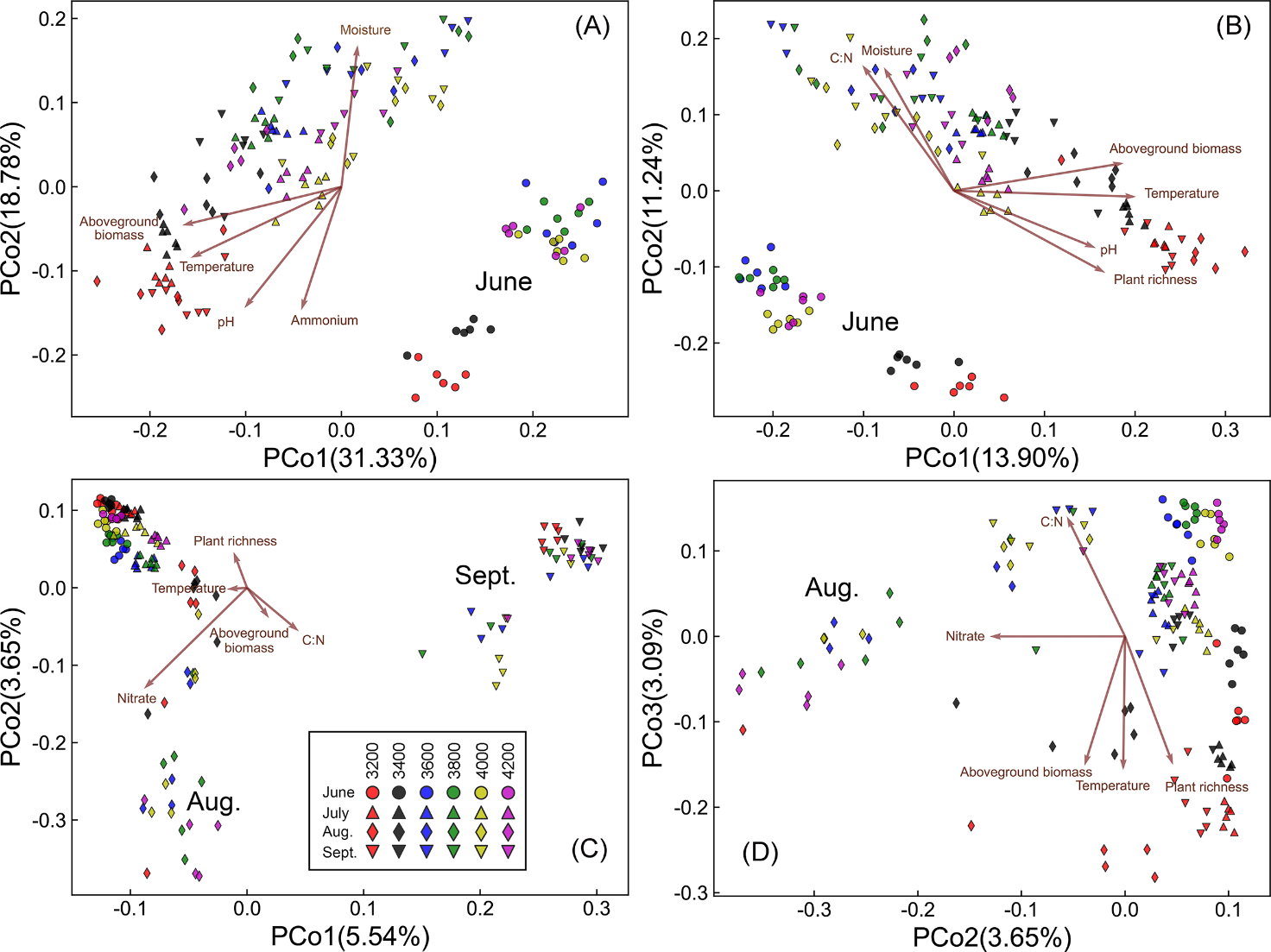


**Supplementary Figure S12.** PCoA plots of prokaryotic subcommunity structures. (A) Abundant species (Axis 1 *vs*. axis 2); (B) Intermediate species (Axis 1 *vs*. axis 2); (C) Rare species (Axis 1 *vs*. axis 2); (D) Rare species (axis 2 *vs*. axis 3). Important environmental factors were fitted onto the patterns. Values on axes indicated the percentages of total variation explained by each axis.


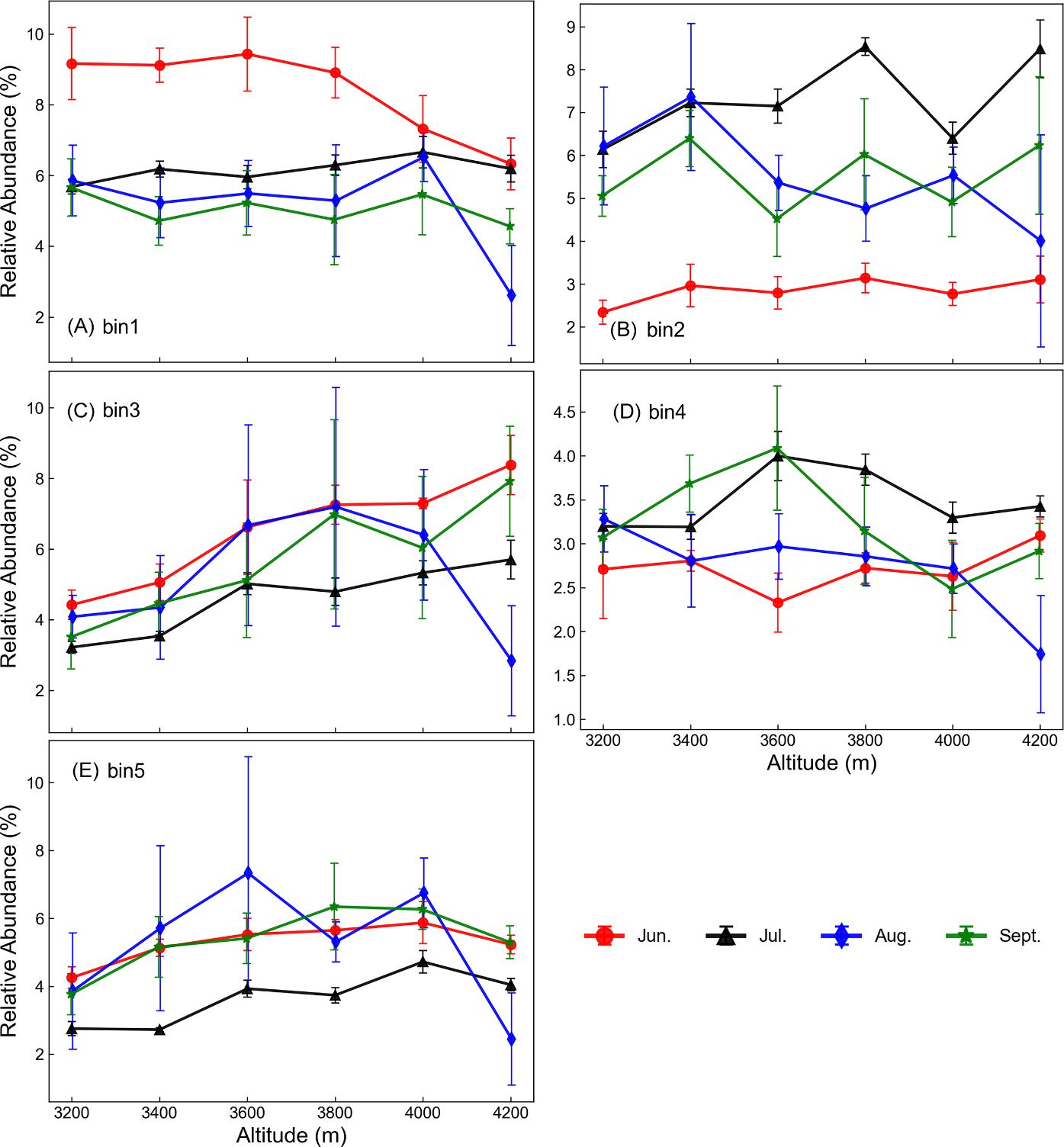


**Supplementary Figure S13.** Relative abundances of iCAMP bins belong to abundant subcommunities. Error bars represent standard deviations (n = 6).

## Supplementary Tables

**Supplementary Table S1.** Spearman's correlation of environmental factors to representative genera with different elevational patterns.

| Elevational pattern | Genus | Ammonium | Nitrate | Nitrite | TN | C:N | pH | Moisture | Precipitation^a^ | Temperature | Plant richness | Aboveground biomass | Altitude |
| --- | --- | --- | --- | --- | --- | --- | --- | --- | --- | --- | --- | --- | --- |
| I | *Nitrospira* | -0.460*** | -0.135 | -0.318*** | -0.191* | 0.302*** | -0.591*** | 0.312*** | -0.611** | -0.769*** | -0.460*** | -0.688*** | 0.547*** |
| I | *Chthoniobacter* | -0.336*** | -0.118 | -0.344*** | -0.354*** | 0.004 | -0.055 | -0.231** | 0.565** | -0.568*** | -0.366*** | -0.394*** | 0.451*** |
| I | *Terrimicrobium* | -0.427*** | 0.047 | 0.024 | -0.230** | 0.272** | -0.089 | 0.088 | 0.722*** | -0.390*** | -0.397*** | -0.043 | 0.475*** |
| I | *Bauldia* | -0.439*** | 0 | -0.114 | -0.291*** | 0.396*** | -0.318*** | 0.281*** | -0.291 | -0.489*** | -0.492*** | -0.245** | 0.591*** |
| I | *Rhodomicrobium* | -0.252** | 0.243** | 0.317*** | -0.084 | 0.327*** | 0.054 | 0.256** | -0.197 | 0.065 | -0.234** | 0.311*** | 0.277*** |
| I | *Rhodoplanes* | -0.231** | -0.145 | -0.385*** | -0.241** | 0.256** | -0.304*** | 0.026 | 0.022 | -0.506*** | -0.312*** | -0.499*** | 0.394*** |
| II | *Arthrobacter* | 0.138 | 0.233** | 0.273** | 0.022 | 0.095 | 0.284*** | 0.011 | 0.598** | 0.500*** | 0.203* | 0.692*** | -0.280*** |
| II | *Oryzihumus* | 0.307*** | 0.073 | 0.179* | -0.051 | -0.045 | 0.374*** | -0.112 | 0.584** | 0.651*** | 0.523*** | 0.611*** | -0.492*** |
| II | *Blastococcus* | 0.252** | 0.353*** | 0.444*** | 0.203* | -0.069 | 0.359*** | 0.002 | -0.194 | 0.801*** | 0.445*** | 0.670*** | -0.505*** |
| II | *Mycobacterium* | 0.306*** | 0.148 | 0.358*** | 0.071 | -0.091 | 0.396*** | -0.081 | 0.361 | 0.681*** | 0.467*** | 0.669*** | -0.495*** |
| II | *Mesorhizobium* | 0.458*** | 0.048 | 0.259** | 0.073 | -0.218** | 0.468*** | -0.209* | 0.393 | 0.715*** | 0.575*** | 0.527*** | -0.623*** |
| II | *Rhizobium* | 0.376*** | -0.007 | 0.180* | 0.024 | -0.088 | 0.471*** | -0.186* | 0.676*** | 0.527*** | 0.435*** | 0.582*** | -0.453*** |
| II | *Devosia* | 0.478*** | -0.203* | -0.024 | -0.095 | -0.226** | 0.507*** | -0.399*** | 0.593** | 0.436*** | 0.478*** | 0.355*** | -0.484*** |
| II | *Lysobacter* | 0.516*** | -0.144 | -0.048 | 0.1 | -0.549*** | 0.403*** | -0.475*** | 0.749*** | 0.391*** | 0.543*** | 0.105 | -0.587*** |
| III | *Candidatus Solibacter* | -0.15 | -0.036 | -0.129 | 0.092 | 0.043 | -0.576*** | 0.319*** | -0.213 | -0.504*** | -0.322*** | -0.525*** | 0.201* |
| III | *Conexibacter* | -0.136 | 0.555*** | 0.453*** | 0.163 | 0.166* | 0.022 | 0.267** | -0.224 | 0.490*** | 0.02 | 0.535*** | -0.028 |
| III | *Nitrososphaera* | -0.248** | -0.057 | 0.048 | -0.224** | 0.377*** | -0.089 | 0.266** | 0.749*** | -0.119 | -0.132 | 0.109 | 0.119 |
| III | *Hyphomicrobium* | -0.282*** | 0.330*** | 0.351*** | 0.114 | 0.436*** | -0.283*** | 0.542*** | -0.396 | 0.112 | -0.192* | 0.238** | 0.157 |
| III | *Defluviicoccus* | -0.249** | 0.273*** | 0.297*** | 0.157 | 0.184* | -0.392*** | 0.543*** | -0.345 | 0.029 | -0.192* | 0.077 | 0.031 |
| III | *Phaselicystis* | -0.211* | 0.032 | 0.077 | 0.166* | 0.281*** | -0.417*** | 0.434*** | -0.815*** | -0.251** | -0.227** | -0.173* | 0.181* |
| IV | *Blastocatella* | 0.176* | -0.344*** | -0.334*** | -0.14 | -0.207* | 0.172* | -0.370*** | 0.253 | -0.166* | 0.04 | -0.297*** | -0.096 |
| IV | *Aridibacter* | 0.025 | -0.270** | -0.422*** | -0.181* | -0.152 | 0.055 | -0.379*** | 0.213 | -0.350*** | -0.104 | -0.502*** | 0.109 |
| IV | *Gemmatimonas* | 0.199* | -0.363*** | -0.426*** | -0.434*** | -0.112 | 0.377*** | -0.486*** | 0.463* | -0.003 | 0.301*** | -0.001 | -0.206* |
| IV | *Aquicella* | 0.300*** | -0.184* | -0.277*** | 0.003 | -0.357*** | 0.119 | -0.402*** | -0.129 | -0.07 | 0.229** | -0.312*** | -0.166* |
| IV | *Ferruginibacter* | 0.122 | -0.373*** | -0.437*** | -0.166* | -0.179* | 0.03 | -0.354*** | -0.673*** | -0.378*** | -0.062 | -0.537*** | 0.142 |
| IV | *Terrimonas* | 0.247** | -0.607*** | -0.370*** | -0.16 | -0.114 | 0.096 | -0.290*** | -0.259 | -0.372*** | 0.03 | -0.455*** | -0.01 |
| V | *Methylibium* | -0.063 | 0.08 | -0.183* | -0.260** | -0.137 | 0.006 | -0.167* | 0.148 | -0.046 | -0.005 | -0.108 | 0.073 |
| V | *Massilia* | -0.16 | 0.024 | -0.227** | -0.379*** | 0.074 | -0.007 | -0.122 | 0.305 | -0.042 | 0.004 | 0.067 | 0.116 |
| V | *Ilumatobacter* | -0.002 | 0.282*** | 0.078 | 0.164 | -0.094 | -0.079 | -0.028 | -0.858*** | 0.07 | 0.019 | -0.153 | 0.091 |
| V | *Pseudonocardia* | 0.083 | 0.402*** | 0.461*** | 0.131 | -0.016 | 0.286*** | 0.042 | 0.129 | 0.578*** | 0.210* | 0.678*** | -0.190* |
| V | *Solirubrobacter* | -0.145 | 0.489*** | 0.367*** | 0.023 | 0.215* | 0.170* | 0.147 | 0.005 | 0.460*** | 0.039 | 0.547*** | 0.015 |
| V | *Nocardioides* | 0.102 | 0.378*** | 0.336*** | 0.190* | -0.003 | 0.176* | 0.016 | -0.313 | 0.530*** | 0.219** | 0.419*** | -0.198* |
| V | *Bradyrhizobium* | 0.170* | 0.06 | 0.279*** | 0.03 | 0.038 | 0.277*** | -0.009 | 0.366 | 0.466*** | 0.235** | 0.538*** | -0.279*** |
| V | *Rhizomicrobium* | 0.168* | -0.262** | -0.345*** | 0.047 | -0.260** | -0.178* | -0.156 | -0.24 | -0.300*** | -0.026 | -0.514*** | 0.016 |

Significance: * P<0.05 (green), ** P<0.01 (yellow), *** P<0.001 (pink). The highest correlation coefficient of each row is in bold and red color.

a. Only data at 3200 m were used.

**Supplementary Table S2.** Spearman's correlation of environmental factors to alpha diversity indices and important taxa.

|  | Ammonium | Nitrate | Nitrite | TN | C:N | pH | Moisture | Precipitation^a^ | Temperature | Plant richness | Aboveground biomass | Altitude |
| --- | --- | --- | --- | --- | --- | --- | --- | --- | --- | --- | --- | --- |
| (1) alpha diversiy |  |  |  |  |  |  |  |  |  |  |  |  |
| Observed species | 0.241** | 0.139 | -0.087 | 0.173* | -0.260** | 0.057 | -0.224** | -0.689*** | 0.198* | 0.190* | -0.131 | -0.165* |
| Chao1 index | 0.142 | 0.242** | -0.019 | 0.190* | -0.217** | -0.025 | -0.12 | -0.695*** | 0.195* | 0.157 | -0.13 | -0.104 |
| Shannon diversity | 0.381*** | 0.076 | -0.042 | 0.117 | -0.279*** | 0.187* | -0.229** | -0.741*** | 0.376*** | 0.362*** | 0.057 | -0.369*** |
| Reciprocal Simpson diversity | 0.444*** | 0.127 | 0.103 | 0.1 | -0.277*** | 0.264** | -0.134 | -0.610** | 0.591*** | 0.525*** | 0.287*** | -0.557*** |
| Pielou evenness | 0.464*** | 0.016 | 0.016 | 0.076 | -0.291*** | 0.310*** | -0.230** | -0.625** | 0.505*** | 0.459*** | 0.261** | -0.511*** |
| (2) abundant phyla |  |  |  |  |  |  |  |  |  |  |  |  |
| Proteobacteria | 0.386*** | -0.315*** | -0.127 | -0.013 | -0.267** | 0.012 | -0.069 | 0.571** | 0.032 | 0.317*** | -0.088 | -0.396*** |
| Acidobacteria | 0.033 | -0.032 | -0.182* | 0.121 | -0.175* | -0.183* | -0.075 | -0.226 | -0.129 | 0.021 | -0.505*** | -0.053 |
| Actinobacteria | -0.069 | 0.466*** | 0.390*** | 0.062 | 0.152 | 0.231** | 0.087 | -0.113 | 0.497*** | 0.089 | 0.599*** | -0.048 |
| Verrucomicrobia | -0.525*** | -0.086 | -0.222** | -0.208* | 0.270** | -0.403*** | 0.167* | 0.748*** | -0.711*** | -0.544*** | -0.446*** | 0.617*** |
| Bacteroidetes | 0.02 | -0.473*** | -0.346*** | -0.214* | 0.031 | -0.008 | -0.136 | 0.027 | -0.475*** | -0.241** | -0.356*** | 0.244** |
| Gemmatimonadetes | 0.257** | -0.312*** | -0.328*** | -0.390*** | -0.121 | 0.443*** | -0.461*** | 0.555** | 0.155 | 0.405*** | 0.141 | -0.334*** |
| Planctomycetes | 0.311*** | -0.122 | -0.105 | 0.126 | -0.391*** | 0.279*** | -0.473*** | -0.797*** | 0.250** | 0.331*** | -0.075 | -0.343*** |
| Nitrospirae | 0.027 | -0.096 | -0.133 | 0.143 | -0.081 | -0.264** | 0.033 | -0.732*** | -0.134 | 0.124 | -0.494*** | -0.077 |
| Chloroflexi | -0.04 | 0.377*** | 0.12 | 0.138 | -0.157 | -0.011 | -0.07 | -0.609** | 0.194* | -0.004 | 0.076 | 0.059 |
| Firmicutes | -0.086 | 0.064 | 0.161 | -0.034 | 0.192* | 0.017 | 0.211* | 0.657*** | 0.044 | -0.059 | 0.404*** | 0.024 |
| Thaumarchaeota | -0.248** | -0.057 | 0.047 | -0.225** | 0.377*** | -0.089 | 0.266** | 0.749*** | -0.118 | -0.132 | 0.109 | 0.118 |
| (3) proteobacterial classes |  |  |  |  |  |  |  |  |  |  |  |  |
| Alphaproteobacteria | 0.425*** | -0.068 | 0.218** | 0.148 | -0.215* | 0.339*** | -0.109 | 0.194 | 0.502*** | 0.404*** | 0.385*** | -0.502*** |
| Betaproteobacteria | -0.173* | -0.196* | -0.284*** | -0.272** | 0.142 | -0.319*** | 0.121 | 0.495* | -0.426*** | -0.149 | -0.339*** | 0.183* |
| Deltaproteobacteria | 0.467*** | -0.079 | 0.042 | 0.340*** | -0.433*** | -0.032 | -0.052 | -0.754*** | 0.254** | 0.389*** | -0.118 | -0.500*** |
| Gammaproteobacteria | 0.389*** | -0.409*** | -0.099 | -0.045 | -0.196* | 0.226** | -0.229** | 0.721*** | 0.021 | 0.233** | -0.001 | -0.287*** |

Significance: * P<0.05 (green), ** P<0.01 (yellow), *** P<0.001 (pink). The highest correlation coefficient of each row is in bold and red color.

a. Only data at 3200 m were used.

**Supplementary Table S3.** The effect of each environmental factor on the community structures composed of abundant, intermediate and rare species, respectively (Permutation: 999).

|  | Ammonium | Nitrate | Nitrite | TN | C:N ratio | pH | Moisture | Temperature | Plant richness | Plant biomass |
| --- | --- | --- | --- | --- | --- | --- | --- | --- | --- | --- |
| (a) Spearman's correlation coefficients using partial Mantel test | | | |  |  |  |  |  |  |  |
| Abundant species | 0.086** | 0.008 | 0.056 | -0.026 | -0.034 | 0.112** | **0.125***** | **0.211***** | 0.035 | **0.250***** |
| Intermediate species | 0.049 | 0.048 | 0.012 | -0.002 | 0.077* | 0.094* | 0.100** | **0.145***** | 0.078** | **0.246***** |
| Rare species | 0.021 | **0.138***** | -0.015 | 0.024 | 0.079** | -0.022 | 0.008 | 0.051 | **0.083***** | **0.131***** |
| (b) Regression coefficients using MRM analysis | | |  |  |  |  |  |  |  |  |
| Abundant species | -0.082** | -0.007 | -0.050 | 0.026 | 0.032 | **-0.117***** | **-0.127***** | **-0.214***** | -0.034 | **-0.247***** |
| Intermediate species | -0.048 | -0.040 | -0.011 | 0.002 | -0.074 | -0.101* | -0.103** | **-0.148***** | -0.079** | **-0.250***** |
| Rare species | -0.022 | **-0.136***** | 0.016 | -0.027 | -0.084** | 0.026 | -0.009 | -0.057* | -0.091** | **-0.141***** |
| (c) Independent contribution using hierarchical partitioning in canonical analysis | | | | |  |  |  |  |  |  |
| Abundant species | 0.040** | 0.024* | 0.023 | **0.027***** | 0.026** | 0.036 | **0.045***** | **0.093***** | 0.039** | **0.090***** |
| Intermediate species | **0.023***** | 0.017** | 0.012 | **0.017***** | **0.016***** | 0.022** | **0.028***** | **0.048***** | 0.024** | **0.043***** |
| Rare species | 0.005** | **0.008***** | 0.002 | **0.006***** | 0.004* | 0.005* | **0.008***** | **0.011***** | 0.005 | **0.010***** |

Significance: * P<0.05, ** P<0.01, *** P<0.001

**Supplementary Table S4.** Relative importance (%) of different ecological processes contributed by each bin of abundant subcommunities.

| Month | Altitude (m) | Process | bin1 | bin2 | bin3 | bin4 | bin5 |
| --- | --- | --- | --- | --- | --- | --- | --- |
| 2021.6 | 3200 | HeS | 0 | 9.56 | 18.11 | 0 | 0 |
| 2021.6 | 3400 | HeS | 0 | 11.84 | 14.93 | 0 | 0 |
| 2021.6 | 3600 | HeS | 0 | 1.47 | 0 | 5.56 | 0 |
| 2021.6 | 3800 | HeS | 0 | 6.98 | 0 | 0 | 0 |
| 2021.6 | 4000 | HeS | 0 | 4.42 | 0 | 0 | 0 |
| 2021.6 | 4200 | HeS | 0 | 7.03 | 0 | 0 | 0 |
| 2021.7 | 3200 | HeS | 14.37 | 0 | 0 | 0 | 0 |
| 2021.7 | 3400 | HeS | 5.49 | 0 | 0 | 0 | 0 |
| 2021.8 | 3200 | HeS | 6.37 | 0 | 0 | 0 | 0 |
| 2021.8 | 3400 | HeS | 14.49 | 0 | 0 | 0 | 2.64 |
| 2021.8 | 3600 | HeS | 0 | 0 | 4 | 0 | 0 |
| 2021.8 | 3800 | HeS | 0 | 0 | 0 | 0 | 3.1 |
| 2021.8 | 4000 | HeS | 4.21 | 0 | 0 | 0 | 0 |
| 2021.8 | 4200 | HeS | 6.51 | 0 | 0 | 0 | 0 |
| 2021.9 | 3200 | HeS | 11.82 | 0 | 1.98 | 0 | 0 |
| 2021.9 | 3400 | HeS | 19.37 | 0 | 0 | 0 | 9 |
| 2021.9 | 3600 | HeS | 0 | 6.21 | 0 | 0 | 0 |
| 2021.9 | 3800 | HeS | 10.86 | 0 | 0 | 0 | 10.17 |
| 2021.9 | 4000 | HeS | 18.37 | 0 | 0 | 0 | 0 |
| 2021.6 | 3200 | HoS | 0 | 0 | 0 | 0 | 14.74 |
| 2021.6 | 3600 | HoS | 0 | 0 | 0 | 0 | 12.73 |
| 2021.6 | 3800 | HoS | 17.38 | 0 | 0 | 0 | 0 |
| 2021.6 | 4000 | HoS | 0 | 0 | 0 | 0 | 3.2 |
| 2021.6 | 4200 | HoS | 0 | 0 | 0 | 0 | 13.25 |
| 2021.7 | 3200 | HoS | 0 | 29.23 | 0 | 0 | 13.12 |
| 2021.7 | 3400 | HoS | 0 | 31.6 | 2 | 11.25 | 0.8 |
| 2021.7 | 3600 | HoS | 0 | 20.13 | 0 | 0 | 10.25 |
| 2021.7 | 3800 | HoS | 0 | 31.4 | 0 | 0 | 0 |
| 2021.7 | 4000 | HoS | 0 | 17.93 | 0 | 3.39 | 7.33 |
| 2021.7 | 4200 | HoS | 0 | 30.44 | 0 | 12.31 | 9.65 |
| 2021.8 | 3200 | HoS | 0 | 26.43 | 0 | 0 | 9.47 |
| 2021.8 | 3400 | HoS | 0 | 14.6 | 0 | 3.59 | 0 |
| 2021.8 | 3600 | HoS | 0 | 0 | 2.76 | 0 | 0 |
| 2021.8 | 3800 | HoS | 0 | 0 | 12.11 | 0 | 0 |
| 2021.8 | 4200 | HoS | 0 | 5.89 | 0 | 2.57 | 0 |
| 2021.9 | 3200 | HoS | 0 | 3.1 | 0 | 0 | 17.87 |
| 2021.9 | 3600 | HoS | 0 | 0 | 6.82 | 0 | 0 |
| 2021.9 | 3800 | HoS | 0 | 0 | 13.19 | 0 | 0 |
| 2021.9 | 4000 | HoS | 0 | 0 | 10.62 | 0 | 0 |
| 2021.9 | 4200 | HoS | 0 | 0 | 23.81 | 10.13 | 0 |
| 2021.6 | 3200 | DL | 40 | 0.67 | 1.21 | 11.85 | 3.86 |
| 2021.6 | 3400 | DL | 36.33 | 0 | 5.24 | 11.18 | 20.48 |
| 2021.6 | 3600 | DL | 35.31 | 9.02 | 24.69 | 3.21 | 8 |
| 2021.6 | 3800 | DL | 14.76 | 4.38 | 26.22 | 9.85 | 20.44 |
| 2021.6 | 4000 | DL | 28.21 | 6.3 | 28.21 | 10.2 | 19.46 |
| 2021.6 | 4200 | DL | 24.17 | 4.85 | 32.07 | 11.86 | 6.78 |
| 2021.7 | 3200 | DL | 12.7 | 0 | 15.34 | 15.23 | 0 |
| 2021.7 | 3400 | DL | 21.54 | 0 | 13.48 | 2.71 | 11.12 |
| 2021.7 | 3600 | DL | 22.86 | 7.31 | 19.27 | 15.34 | 4.84 |
| 2021.7 | 3800 | DL | 23.13 | 0 | 17.62 | 14.12 | 13.74 |
| 2021.7 | 4000 | DL | 25.2 | 6.32 | 20.19 | 9.09 | 10.55 |
| 2021.7 | 4200 | DL | 22.23 | 0 | 20.48 | 0 | 4.88 |
| 2021.8 | 3200 | DL | 18.9 | 0 | 17.73 | 14.24 | 6.86 |
| 2021.8 | 3400 | DL | 6.21 | 14.18 | 17.43 | 7.44 | 19.42 |
| 2021.8 | 3600 | DL | 19.99 | 19.61 | 16.81 | 10.8 | 26.02 |
| 2021.8 | 3800 | DL | 20.48 | 19.55 | 15.09 | 11.4 | 18.26 |
| 2021.8 | 4000 | DL | 19.28 | 19.91 | 22.58 | 9.83 | 24.19 |
| 2021.8 | 4200 | DL | 12.22 | 23.07 | 20.27 | 11.15 | 18.31 |
| 2021.9 | 3200 | DL | 15.03 | 20.95 | 14.57 | 14.69 | 0 |
| 2021.9 | 3400 | DL | 0 | 26.19 | 18.24 | 15.09 | 12.12 |
| 2021.9 | 3600 | DL | 21.4 | 12.43 | 14.13 | 16.81 | 22.21 |
| 2021.9 | 3800 | DL | 6.33 | 22.34 | 12.01 | 11.52 | 13.57 |
| 2021.9 | 4000 | DL | 3.23 | 19.7 | 13.02 | 9.9 | 25.15 |
| 2021.9 | 4200 | DL | 16.98 | 23.06 | 5.6 | 0.71 | 19.72 |

HeS: heterogeneous selection; HoS: homogeneous selection; DL: dispersal limitation.

The abundant sub-communities included 5 bins:

bin1: mainly Acidobacteria (classes Blastocatellia and Gp16).

bin2: Actinobacteria.

bin3: Acidobacteria (classes Vicinamibacteria and Holophagae) and Verrucomicrobia.

bin4: Alphaproteobacteria, such as *Bradyrhizobium* and *Mesorhizobium*.

bin5: mainly Betaproteobacteria, such as Nitrosomonadaceae.
